# Biallelic variants in *FLII* cause pediatric cardiomyopathy by disrupting cardiomyocyte cell adhesion and myofibril organization

**DOI:** 10.1172/jci.insight.168247

**Published:** 2023-09-08

**Authors:** Claudine W.B. Ruijmbeek, Filomena Housley, Hafiza Idrees, Michael P. Housley, Jenny Pestel, Leonie Keller, Jason K.H. Lai, Herma C. van der Linde, Rob Willemsen, Janett Piesker, Zuhair N. Al-Hassnan, Abdulrahman Almesned, Michiel Dalinghaus, Lisa M. van den Bersselaar, Marjon A. van Slegtenhorst, Federico Tessadori, Jeroen Bakkers, Tjakko J. van Ham, Didier Y.R. Stainier, Judith M.A. Verhagen, Sven Reischauer

**Affiliations:** 1 Department of Clinical Genetics, Erasmus MC, University Medical Center Rotterdam, Rotterdam, Netherlands.; 2 Department of Developmental Genetics, Max Planck Institute for Heart and Lung Research, Bad Nauheim, Germany.; 3 Medical Clinic I (Cardiology/Angiology) and Campus Kerckhoff, Justus-Liebig-University Giessen, Giessen, Germany.; 4 Excellence Cluster Cardio-Pulmonary Institute (CPI), Giessen/Bad Nauheim, Germany.; 5 Scientific Service Group Microscopy, Max Planck Institute for Heart and Lung Research, Bad Nauheim, Germany.; 6 Department of Medical Genetics, and; 7 Cardiovascular Genetics Program, King Faisal Specialist Hospital & Research Center, Riyadh, Saudi Arabia.; 8 Prince Sultan Cardiac Center, Buraidah, Saudi Arabia.; 9 Department of Pediatric Cardiology, Erasmus MC, University Medical Center Rotterdam, Rotterdam, Netherlands.; 10 Hubrecht Institute-KNAW and University Medical Center Utrecht, Utrecht, Netherlands.; 11 Department of Pediatric Cardiology, University Medical Center Utrecht, Utrecht, Netherlands.; 12 German Centre for Cardiovascular Research (DZHK), RheinMain partner site, Bad Nauheim, Germany.

**Keywords:** Cardiology, Genetics, Cardiovascular disease, Embryonic development, Molecular genetics

## Abstract

Pediatric cardiomyopathy (CM) represents a group of rare, severe disorders that affect the myocardium. To date, the etiology and mechanisms underlying pediatric CM are incompletely understood, hampering accurate diagnosis and individualized therapy development. Here, we identified biallelic variants in the highly conserved flightless-I (*FLII*) gene in 3 families with idiopathic, early-onset dilated CM. We demonstrated that patient-specific *FLII* variants, when brought into the zebrafish genome using CRISPR/Cas9 genome editing, resulted in the manifestation of key aspects of morphological and functional abnormalities of the heart, as observed in our patients. Importantly, using these genetic animal models, complemented with in-depth loss-of-function studies, we provided insights into the function of Flii during ventricular chamber morphogenesis in vivo, including myofibril organization and cardiomyocyte cell adhesion, as well as trabeculation. In addition, we identified Flii function to be important for the regulation of Notch and Hippo signaling, crucial pathways associated with cardiac morphogenesis and function. Taken together, our data provide experimental evidence for a role for FLII in the pathogenesis of pediatric CM and report biallelic variants as a genetic cause of pediatric CM.

## Graphical abstract

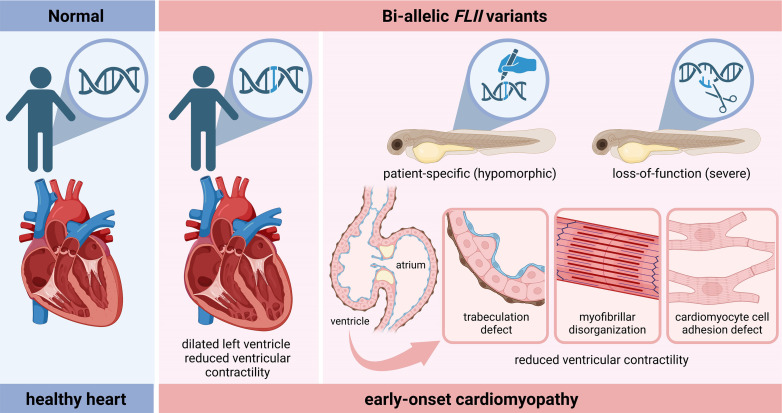

## Introduction

Pediatric cardiomyopathy (CM) corresponds to a group of clinically and genetically heterogeneous structural and functional disorders affecting the myocardium. Pediatric CM is estimated to occur in 1 in 100,000 children per year. Dilated CM (DCM), characterized by ventricular dilation and impaired myocardial contractility, is the most prevalent subtype among children (1). The prognosis of pediatric CM is generally poor, especially in DCM, as approximately half of the children require cardiac transplantation or die from cardiac complications within the first years after diagnosis (2). The understanding of the genetic basis of pediatric CM has been significantly improved by the advent of next-generation genomic sequencing (3). In fact, variants in more than 100 genes that belong to various molecular pathways involved in myocardial contraction, energy metabolism, and calcium handling, among others, have been linked to pediatric CM. However, most of the reported associations are rare, and only a few have been experimentally confirmed. Consequently, to date, more than half of pediatric CM cases remain idiopathic (3). Given the marked genetic heterogeneity of pediatric CM, it is anticipated that multiple important causal genes and their underlying pathogenic mechanisms await discovery. Obtaining a genetic diagnosis and dissecting underlying disease-causing mechanisms are important in order to guide early treatment and to develop new therapeutic strategies to improve the prognosis of affected individuals.

Previously, genetic analysis in a consanguineous population revealed a diverse group of candidate genes for pediatric CM, encoding metabolic enzymes, transcription factors, regulators of autophagy, and structural proteins, notably including flightless-I (FLII) (4). In the present study, family-based whole-exome sequencing in a nonconsanguineous Dutch family with early-onset DCM revealed biallelic variants in the *FLII* gene. Together, all the identified variants affected evolutionarily conserved residues and were classified as deleterious using in silico predictions. FLII, a member of the gelsolin superfamily, was initially described to be involved in the regulation of actin dynamics (5). In addition, FLII has been reported to localize at cell adhesion sites, and its deficiency affects cell adhesion complex formation and maturation, consequently influencing cell migration (6–8). Gene knockout of *FLII* homologs leads to embryonic lethality in *Drosophila*, zebrafish, and mouse (9–12). In *Drosophila*, multiple alleles have been described that affect early embryonic cellularization as well as indirect flight muscle development (13). Similarly, zebrafish *flii* mutations cause skeletal muscle fiber disorganization and burst swimming disability (11, 14). These data strongly suggest that FLII plays a crucial role in striated muscle function. However, the precise role of FLII in cardiomyocytes remains poorly understood, and there is scarce evidence regarding the potential association between FLII variants and human cardiac disease (4, 12).

This report provides experimental evidence for a role for FLII in cardiomyocytes and in the pathogenesis of early-onset DCM. Using CRISPR/Cas9-mediated genome editing in zebrafish, we functionally verified the pathogenicity of the discovered genetic variants in *FLII*. In addition, our findings highlight an essential role for Flii in myofibril organization and cardiomyocyte cell adhesion sites during ventricular chamber morphogenesis as well as the regulation of Notch and Hippo signaling, pathways that are crucial in regulating early cardiac development.

## Results

### Clinical and molecular patient characteristics.

**Figure 1:**
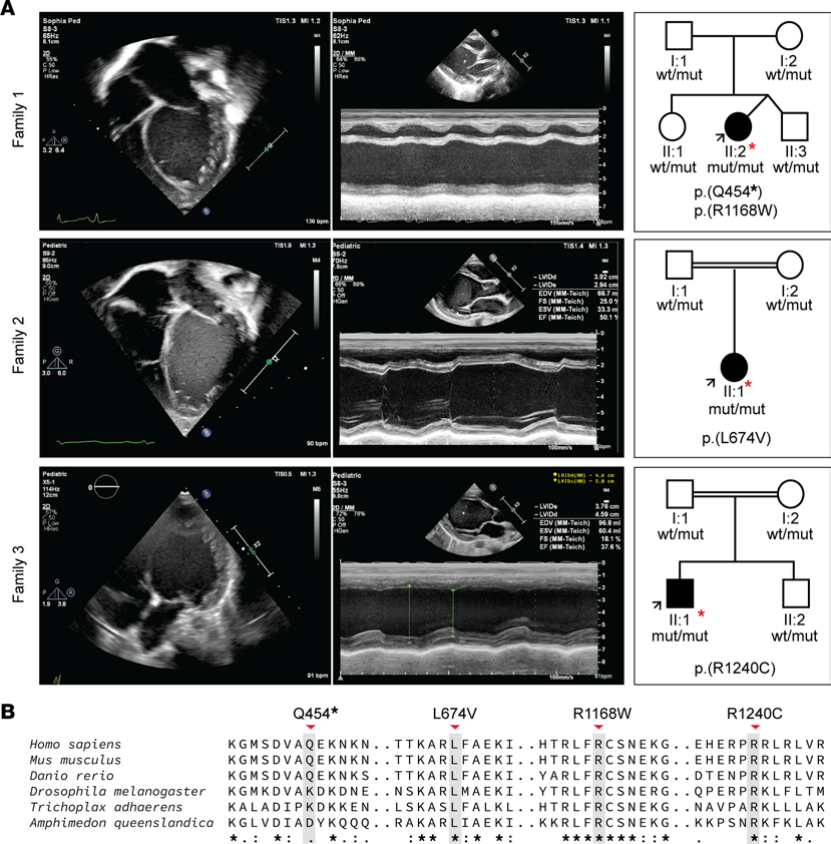
Clinical manifestation of early-onset DCM and family pedigree of affected individuals included in this study. (**A**) Echo findings at presentation. Left panel, per family: 2-dimensional, apical 4-chamber echocardiographic image of the probands depicting an enlarged and spherically shaped left ventricle. Middle panel: M-mode echocardiography displaying severely depressed left ventricular (LV) function. Right panel: family pedigrees that were found to segregate biallelic variants in the *FLII* gene. I and II refer to the first and second generations of the family, respectively. The arrow points to the proband. (**B**) Alignment of FLII protein sequence across the metazoan kingdom including orthologs from invertebrates and vertebrates. Note the full conservation of the affected amino acid residues and high contextual conservation in a wide range of species ranging from simple multicellular organisms including sponges (*Amphimedon queenslandica*) and placozoa (*Trichoplax adhaerens*) to higher species including insects (*Drosophila melanogaster*), bony fishes (*Danio rerio*), rodents (*Mus musculus*), and primates (*Homo sapiens*) illustrating functional significance.

We identified 3 unrelated patients with early-onset DCM and biallelic variants in the *FLII* gene, including 1 nonconsanguineous family of Dutch ancestry and 2 consanguineous families of Saudi Arabian ancestry (Figure 1A). The latter 2 families have been described in a previous cohort study (family 2 = D-151, family 3 = D-071) (4). All patients presented with signs of DCM within the first year of life (age range: 2–5 months) with severely reduced left ventricular ejection fraction (LVEF) (range 23%–32%). Patient 2-II:1 also displayed a secondary atrial septal defect. Aside from initial tachycardia in patient 1-II:2, none of the children displayed signs of arrhythmias (Supplemental Figure 1, A–C; supplemental material available online with this article; https://doi.org/10.1172/jci.insight.168247DS1). No additional extracardiac features were detected. At last follow-up (age range 2–9 years), all patients were alive and showed either stable disease or improved cardiac function. Each of the parents was heterozygous for one of the *FLII* variants. None of the parents showed clinical signs of DCM at cardiac screening. Clinical details of all probands are provided in Table 1 and Supplemental Table 1. All detected *FLII* variants were present in the heterozygous state at a very low frequency or absent from the gnomAD v2.1.1 (Table 1): 1 of the variants was predicted to result in a premature stop codon, and the other 3 variants were missense, affecting highly conserved amino acids (Figure 1B), and were predicted to be damaging by in silico algorithms (Alamut Visual Plus software) but not to have a major effect on the nearest mRNA splice sites.

### Genome-edited zebrafish harboring patient-specific variants display functional and morphological abnormalities of the ventricular myocardium.

To obtain genetic evidence for the pathogenicity of the discovered rare variants, and to understand the role of FLII in cardiac function and disease, we employed the zebrafish vertebrate model system, which is widely used to study cardiac development and function and to model human cardiovascular diseases (15–17). The single zebrafish *flii* homolog encodes a 1,259–amino acid protein with 82.68% sequence identity to the human FLII protein. Computational 3D modeling of both FLII homologs also revealed high conservation at the structural level (Supplemental Figure 2A), suggesting functional conservation. In zebrafish, we found that *flii* was expressed throughout all stages of embryonic development (Supplemental Figure 2B), which is in line with publically available data (18). Moreover, single-cell RNA-sequencing (scRNA-Seq) data from both human and zebrafish cardiac tissue indeed revealed a uniform and widespread distribution of *FLII* expression, like other genes involved in cardiac function and disease (Supplemental Figure 2, C and D). This further supports the notion that the function of FLII in cardiac tissue is likely to be conserved across these species. In adult zebrafish myocardial tissue, we found Flii to be present in ventricular cardiomyocytes, where it particularly localized to cardiomyocyte cell-cell adhesion structures (intercalated disks) and cardiomyocyte cell-matrix adhesions (costamere-like structures) (Supplemental Figure 2E; yellow and blue arrows, respectively).

**Figure 2:**
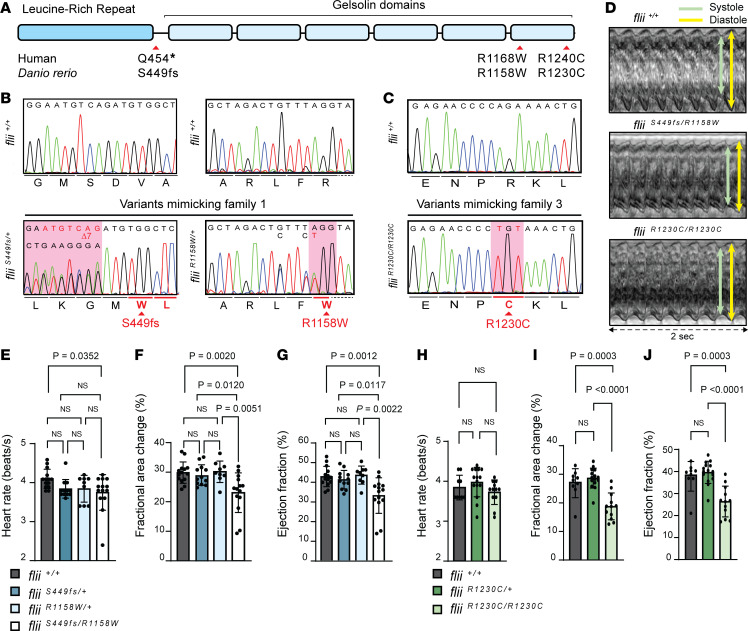
CRISPR/Cas9-mediated genome editing of patient-specific biallelic variants in *flii* in zebrafish results in DCM-associated phenotypes in early development. (**A**) Schematic representation of the FLII protein, consisting of a leucine-rich repeat (LRR) and 6 gelsolin-like domains. Location of the human variants of families 1 and 3 are depicted with corresponding variants generated in zebrafish (*D*. *rerio*). (**B**) Aligned Sanger sequencing traces of wild-type *flii^+/+^* and genome-edited PCR amplicons from compound heterozygous *flii^S449fs/R1158W^* larvae harboring a heterozygous variant resulting in a frameshift starting at amino acid position 449 (left panel, sequence is reverse complement as it was sequenced with the reverse primer) and the heterozygous R1158W missense variant (right panel), representing family 1. Modified codons are underlined in red. Dotted line represents intronic sequence. (**C**) Representative Sanger sequencing results of the PCR amplicons from wild-type and *flii^R1230C/R1230C^* zebrafish larvae harboring the homozygous R1230C missense variant, representing family 3. The modified codon is underlined in red. (**D**) Ventricular kymographs derived from high-speed imaging video recordings of 120 hpf zebrafish larvae spanning approximately 2 seconds: *flii^+/+^* (top panel), *flii^S449fs/R1158W^* (middle panel) and *flii^R1230C/R1230C^* (lower panel). Note that there are no signs of irregular heart rhythm in larvae harboring patient-specific biallelic variants. (**E**–**G**) Ventricular contractility parameters derived from high-speed imaging movies, including heart rate (**E**), fractional area change (**F**), and ejection fraction (**G**) for *flii^+/+^*, *flii^S449fs/+^*, *flii^R1158W/+^*, and *flii^S449fs/R1158W^*. *flii^+/+^* *n* = 14; *flii^S449fs/+^* *n* = 12; *flii^R1158W/+^* *n* = 9; *flii^S449fs/R1158W^* *n* = 14. (**H**–**J**) Ventricular contractility parameters derived from high-speed imaging movies, including heart rate (**H**), fractional area change (**I**), and ejection fraction (**J**) for *flii^+/+^*, *flii^R1230C/+^*, and *flii^R1230C/R1230C^*. *flii^+/+^* *n* = 9; *flii^R1230C/+^* *n* = 15; *flii^R1230C/R1230C^* *n* = 12. Statistics: mean ± SD; 1-way ANOVA coupled with Tukey’s multiple-comparison test was used to test for significance.

To investigate the pathogenicity of the discovered rare variants, nucleotide modifications mimicking the human variants were introduced into the zebrafish *flii* locus (Figure 2A) using CRISPR/Cas9 genome editing (19, 20). To mimic the stop variant p.(Q454*) of family 1, a 7 bp deletion was created in exon 12 by introducing a premature stop codon after amino acid position 449, referred to as *flii^S449fs^* (Supplemental Figure 3A). Next, the missense variants p.(R1168W) of family 1 and p.(R1240C) of family 3, corresponding to R1158W (*flii^R1158W^*; Supplemental Figure 3B) and R1230C (*flii^R1230C^*; Supplemental Figure 3C), respectively, were introduced in zebrafish. Compound heterozygous progeny (Figure 2B) were obtained by mating *flii^R1158W/+^* zebrafish with *flii^S449fs/+^* zebrafish, and homozygous *flii^R1230C/R1230C^* (Figure 2C) larvae were obtained from *flii^R1230C/+^* intercrosses. *flii^S449fs/R1158W^* and *flii^R1230C/R1230C^* larvae were obtained in normal Mendelian ratios, did not differ morphologically from *flii* wild-type and heterozygous siblings at 120 hours postfertilization (hpf) (Supplemental Figure 4, A–C), and were viable through adulthood. Examination of *flii* expression among homozygous missense mutants revealed no signs of mRNA degradation (Supplemental Figure 5A), suggesting that the mutated mRNA can be translated into protein. Computational 3D modeling of missense variants predicted profound alterations in the protein’s tertiary structure. These include the disruption of hydrogen bonds and changes in its 3D folding (Supplemental Figure 5, B–E, and Supplemental Video 1), with potential functional consequences. Notably, the predictions for both wild-type and mutant FLII were strikingly similar between the 2 species, further supporting the suitability of the zebrafish model system to investigate the effect of the specific variants.

To assess whether the introduced variants in Flii lead to detectable functional phenotypic variations in the heart, high-speed video imaging of the beating embryonic hearts and subsequent functional image analysis were performed (20, 21) (Supplemental Videos 2–4, and Figure 2D). The heart rate of 120 hpf mutant larvae harboring patient-specific *flii* variants was similar to that of wild-type larvae, and there were no signs of irregular heart rhythms (Figure 2, D, E, and H). Although no significant differences were detected in mean end-diastolic volume (EDV) (Supplemental Figure 6, A and C) and end-systolic volume (ESV) per genotype (Supplemental Figure 6, B and D), patient-specific larvae displayed significantly reduced ventricular contractility, as indicated by a decreased fractional area change (FAC) (Figure 2, F and I) and EF (Figure 2, G and J).

**Figure 3:**
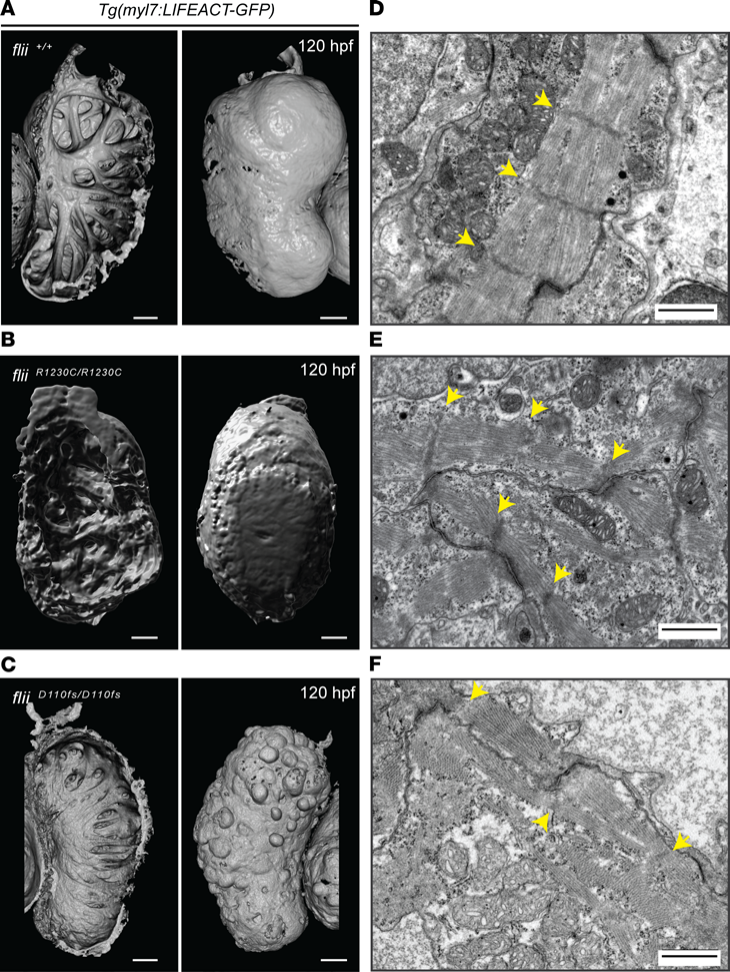
Flii dysfunction results in myofibrillar architectural abnormalities of the ventricular myocardium. (**A**–**C**) 3D volume renderings of maximum projections of *Tg(myl7:LIFEACT-GFP)* cardiac ventricles at 120 hpf from wild-type *flii^+/+^* (**A**), patient-specific *flii^R1230C/R1230C^* (**B**), and *flii^D110fs/D110fs^* (**C**); left panels show ventricular lumen; right panels show ventricular surface. Note that the complex trabecular network observed in wild-type is affected in both mutant alleles. In the severe loss-of-function *flii^D110fs^* mutants, some of the epithelial shaped cardiomyocytes adopt a spherical shape and blebb out of the ventricular wall. Scale bars: 50 μm. Sample size for each genotype, *n* ≥ 3 biological replicates. (**D**–**F**) Representative TEM images of ventricular cardiac muscle from 120 hpf larvae, showing well-organized bundled myofibrils and z-discs in wild-type *flii^+/+^* (**D**), which are disorganized in patient-specific *flii^R1230C/R1230C^* mutants (**E**) and appear to be more severely affected in *flii^D110fs/D110fs^* mutants with faintly present z-discs (**F**). Yellow arrows, z-discs. Scale bars, 1 μm. Sample size for each genotype, *n* ≥ 3 biological replicates.

The question next addressed was whether the reduced ventricular contractility in zebrafish models mimicking 2 independent *FLII* genotypes of patients corresponded to a disorganization of the ventricular myofibrils, given the presence of Flii in intercalated discs and costamere-like structures. For these experiments, one of the generated biallelic zebrafish genotypes, the *flii^R1230C/R1230C^* mutant, was examined in depth. As the disease-causing mechanism of the biallelic *flii* variants was expected to be hypomorphic, resulting in reduced protein function and subtle phenotypic differences, the previously published *flii^D110fs/D110fs^* mutant line, which harbors a homozygous premature stop mutation in exon 5 of the *flii* gene (11), was also investigated. Accordingly, *flii^D110fs/D110fs^* animals lack substantial parts of the Flii protein, including functional gelsolin domains (Supplemental Figure 7). Wild-type and mutant lines were crossed into the *Tg(myl7:LIFEACT-GFP)* reporter line, which fluorescently labels myocardial F-actin to allow visualization of the myofibrillar architecture (22). Examination of 3D-rendered confocal projections of cardiac ventricles revealed that distinct and interconnected trabeculae were prominent in the ventricular lumen of wild-type larvae (Figure 3A, left panel). In contrast, *flii^R1230C/R1230C^* larvae exhibited less organized and more primitive myocardial trabeculae (Figure 3B, left panel), whereas only a few trabeculae could be detected in the ventricular lumen of *flii^D110fs/D110fs^* larvae (Figure 3C, left panel). In addition to the severe reduction in ventricular wall complexity, *flii^D110fs/D110fs^* larvae also displayed cardiomyocyte extrusions toward the abluminal side of the ventricle (Figure 3C, right panel). These cell-architectural changes were not visible in wild-type larvae or *flii^R1230C/R1230C^* mutants (Figure 3, A and B, right panels). Detailed transmission electron microscopy (TEM) examination verified that wild-type larvae had well-organized, compact, bundled myofibrils with clearly defined z-discs and intercalated discs at 120 hpf (Figure 3D). In contrast, myofibrils and intercalated discs of *flii^R1230C/R1230C^* larvae were less densely packed and irregularly oriented (Figure 3E). *flii^D110fs/D110fs^* larvae displayed even more severely affected myofibrils, with irregularly and poorly defined filament organization (Figure 3F).

### Cardiac myofibril maturation is compromised in Flii-deficient larvae.

To better understand the physiological role of Flii in myocardial development and function, in vivo imaging was utilized to study the Flii-deficient *flii^D110fs/D110fs^* zebrafish mutants. Observations of *flii^D110fs/D110fs^* non-3D-rendered confocal projections revealed myofibril disorganization as early as 72 hpf (Supplemental Figure 8). Higher magnification projections of 72 hpf *flii^D110fs/D110fs^* mutant ventricular surfaces clearly showed the presence of myofibrils that were thinner than those of the wild-type and heterozygous larvae (referred to as *flii^+/?^* siblings) (Supplemental Figure 9, A and B). Interestingly, as also observed in the TEM images (Figure 3, D–F), mutant myofibrils exhibited the characteristic sarcomeric banding pattern but failed to expand in size like the myofibrils of *flii^+/?^* siblings (Supplemental Figure 9C). These results suggest that Flii is dispensable for sarcomere assembly and myofibril formation but appears to be required for myofibril bundling, a process that is dependent on myofibril anchorage to costameres (23).

### Cardiac trabeculation is dependent on Flii.

**Figure 4:**
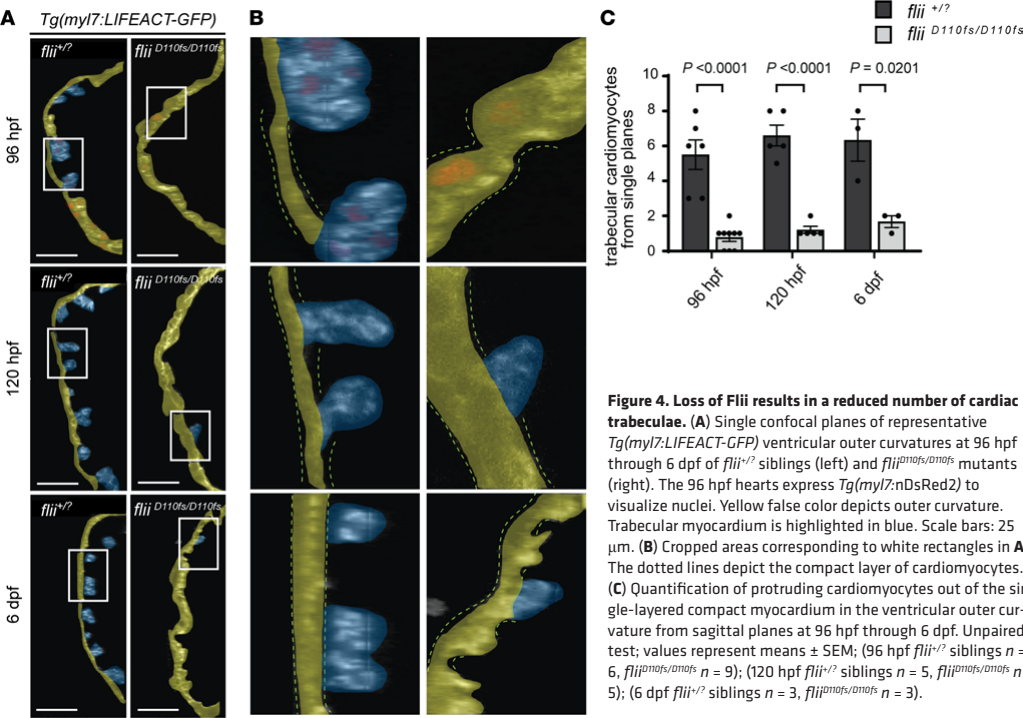
Loss of Flii results in a reduced number of cardiac trabeculae. (**A**) Single confocal planes of representative *Tg(myl7:LIFEACT-GFP)* ventricular outer curvatures at 96 hpf through 6 dpf of *flii^+/?^* siblings (left) and *flii^D110fs/D110fs^* mutants (right). The 96 hpf hearts express *Tg(myl7:*nDsRed2*)* to visualize nuclei. Yellow false color depicts outer curvature. Trabecular myocardium is highlighted in blue. Scale bars: 25 μm. (**B**) Cropped areas corresponding to white rectangles in **A**. The dotted lines depict the compact layer of cardiomyocytes. (**C**) Quantification of protruding cardiomyocytes out of the single-layered compact myocardium in the ventricular outer curvature from sagittal planes at 96 hpf through 6 dpf. Unpaired *t* test; values represent means ± SEM; (96 hpf *flii^+/?^* siblings *n* = 6, *flii^D110fs/D110fs^* *n* = 9); (120 hpf *flii^+/?^* siblings *n* = 5, *flii^D110fs/D110fs^* *n* = 5); (6 dpf *flii^+/?^* siblings *n* = 3, *flii^D110fs/D110fs^* *n* = 3).

Since both the missense *flii^R1230C/R1230C^* larvae harboring a patient-specific *FLII* variant and the *flii^D110fs/D110fs^* mutants displayed abnormal morphology of the ventricular trabecular network at 120 hpf, the question arose as to whether Flii is a regulator of ventricular chamber morphogenesis. In zebrafish, ventricular trabeculation starts between 60 and 65 hpf, when a subset of cardiomyocytes begins to delaminate from the single-layered, compact myocardium (24, 25). During this process, trabeculating cardiomyocytes undergo architectural changes of the cytoskeleton and cell adhesions in order to protrude into the ventricular lumen while maintaining their mechanical function during heart contractions. By 84 hpf, cardiac trabeculation is more pronounced, with trabeculae distributed mostly along the outer curvature of the ventricle (24, 25). Quantification of protruding cardiomyocytes from ventricular outer curvatures indeed showed that their number was reduced in *flii* mutants (Figure 4). These results suggest that Flii is required for trabeculation during ventricular chamber morphogenesis. However, since previous studies have shown the importance of hemodynamic forces during trabeculation (26, 27), we wanted to test whether these observed trabeculation defects were a consequence of changes in blood flow or hemodynamic forces.

**Figure 5:**
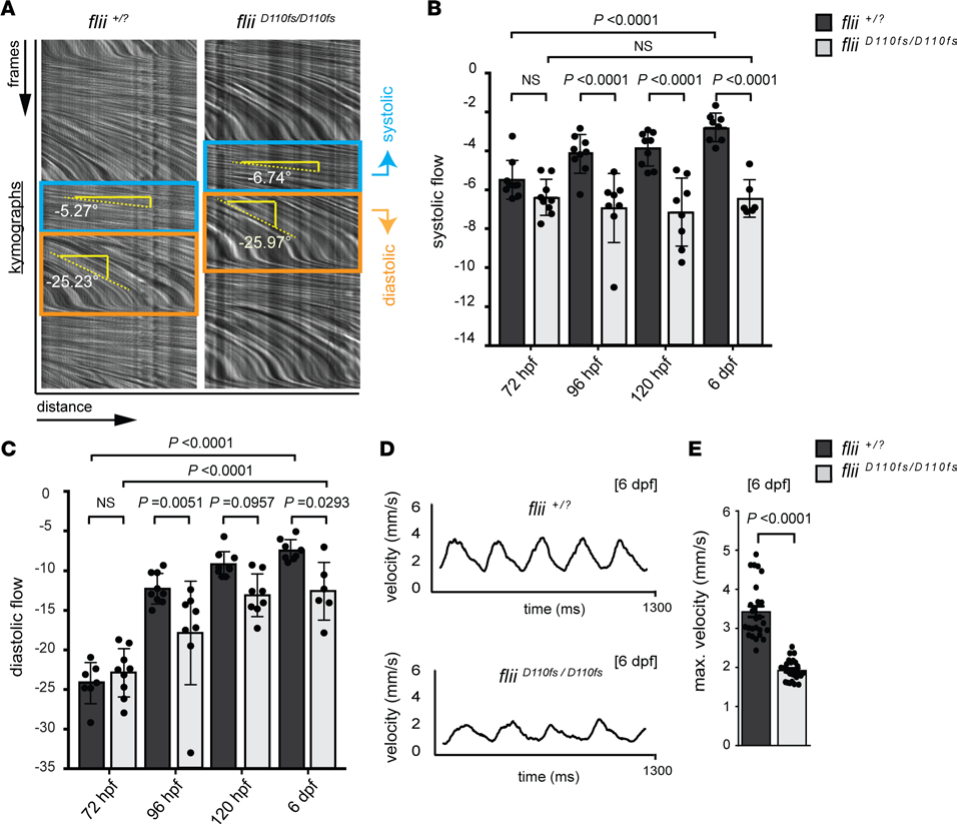
Blood flow analysis reveals reduced cardiac performance upon Flii deficiency, including a developmental arrest in the systolic hemodynamic force. (**A**–**C**) Analysis of blood flow velocity (BFV) in the dorsal aorta by spinning disk microscopy at 72 hpf through 6 dpf. (**A**) Blood flow videos (400 frames/s, total of 500 frames shown) are visualized as kymographs, which show dynamics of blood cells that move distance *x* over frames *y*. Relative speeds are determined by measuring the angle of blood flow in the kymographs, with a steeper downward angle representing slower blood flow. In the systolic phase (blue box), blood cells move faster than in the diastolic phase (orange box). (**B** and **C**) Quantification of kymograph angles in systolic and diastolic phases, respectively. Note that the systolic blood cell speed does not increase in *flii^D110fs^* mutants with developmental time (**B**). In contrast, the diastolic blood cell speed increases in *flii^D110fs^* mutants but is still significantly reduced compared with that of wild-type and heterozygous siblings (**C**). One-way ANOVA coupled with Holm-Šídák multiple-comparison test was used to test for significance; values represent means ± SEM; (72 hpf *flii^+/?^* siblings *n* = 7, *flii^D110fs/D110fs^* *n* = 9); (96 hpf *flii^+/?^* siblings *n* = 9, *flii^D110fs/D110fs^* *n* = 8); (120 hpf *flii^+/?^* siblings *n* = 9, *flii^D110fs/D110fs^* *n* = 8); (6 dpf *flii^+/?^* siblings *n* = 8, *flii^D110fs/D110fs^* *n* = 6). (**D** and **E**) Quantification of absolute blood cell speed by single-cell tracking at 6 dpf reveals a normal sinus rhythm of heartbeats in both *flii^D110fs/D110fs^* and *flii^+/?^* siblings. Bar graphs display maximum velocity of blood cells in the dorsal aorta. Unpaired *t* test; values represent means ± SEM.

To do so, we established a method to measure blood flow velocity (BFV) to assess hemodynamics directly using high-frame-rate recordings of the blood flow in the dorsal aorta (400 frames/s, 500 total frames) that can be converted into kymographs and analyzed (Figure 5A). Notably, at 72 hpf, neither systolic nor diastolic BFV was reduced in *flii^D110fs/D110fs^* mutants compared with those of *flii^+/?^* siblings. Since trabeculation is initiated between 60 and 65 hpf, these results suggest that altered hemodynamics are not the primary cause for the observed trabeculation defects (Figure 5, B and C). From 96 hpf onward, systolic BFV increased with developmental time in *flii^+/?^* sibling larvae (Figure 5B, dark gray bar). In contrast, *flii^D110fs/D110fs^* mutants did not display an increase in systolic BFV between 96 hpf and 6 dpf (Figure 5B, light gray bars). However, diastolic BFV did increase substantially during development in *flii^+/?^* siblings as well as in *flii^D110fs/D110fs^* mutants, though in *flii^D110fs/D110fs^* mutants it was reduced compared with that of *flii^+/?^* siblings (Figure 5C), possibly due to the reduced trabeculation at these developmental stages.

As an independent measure of BFV, the absolute BFV was next measured in the dorsal aorta by tracking individual blood cells. BFV over time followed the characteristic sine wave of cardiac contractions, with cells accelerating during the systolic phase and decelerating during the diastolic phase (Figure 5D). Consistent with the BFV results mentioned above, *flii^D110fs/D110fs^* larvae exhibited a 44% reduction in maximum blood cell velocity at 6 dpf (Figure 5E). As observed in the ventricular contractility measurements in zebrafish models mimicking patient *FLII* variants (Figure 2, E and H), there were no significant changes in the overall heart rate in *flii^D110fs/D110fs^* animals at 72 hpf through 6 dpf (results not shown). Despite the clear cardiomyocyte architectural changes and the lack of bundled myofibrils, *flii^D110fs/D110fs^* mutants did not develop pericardial edema, and their hearts were still contractile at 6 dpf, but *flii^D110fs/D110fs^* mutants did not survive past 10 dpf. Taken together, these results indicate that Flii is an essential regulator of trabeculation during zebrafish ventricular chamber morphogenesis and that Flii dysfunction results in reduced cardiac contractility without affecting the heart rate.

### Flii is essential for the assembly of cardiomyocyte cell adhesion complexes.

Flii localizes at cell adhesion sites, where it colocalizes and binds to the focal adhesion protein vinculin (Vcl) (7, 8, 28). Furthermore, Flii can actively regulate cell adhesion dynamics and cytoskeletal rearrangements, providing further support for its important role at these sites (6–8, 28, 29). In line with these data, immunohistochemistry detected Flii localized at cell adhesion sites (intercalated discs and costamere-like structures) in zebrafish ventricular cardiomyocytes (Supplemental Figure 2E). Both types of structures are highly organized, consisting of membrane-spanning multiprotein complexes that ultimately connect to cytoskeletal F-actin and myofibrils via α-catenin and Vcl, respectively (30).

**Figure 6:**
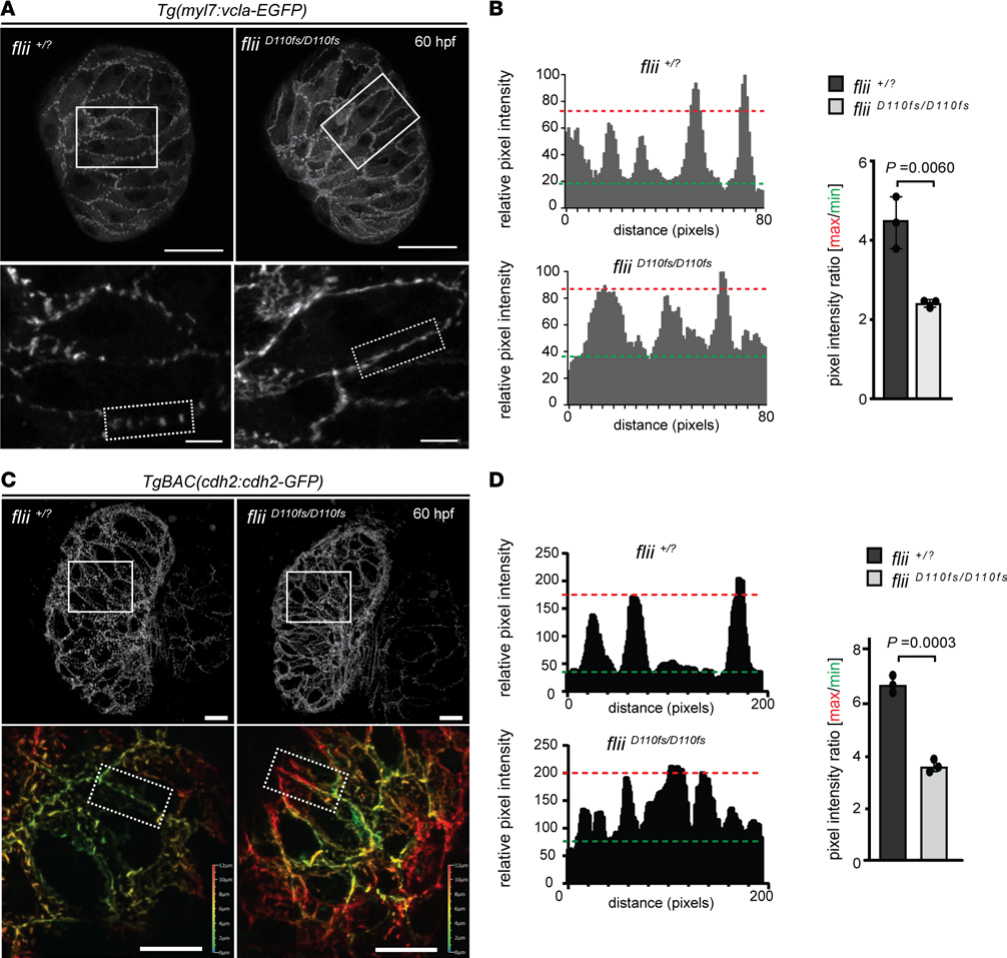
Flii-deficient zebrafish exhibit defects in vinculin-EGFP and cadherin2-EGFP localization. (**A**) 3D confocal projections of 60 hpf *Tg(myl7:vcla-EGFP*) *flii^+/?^* sibling and *flii^D110fs/D110fs^* cardiac ventricles. Vinculin-EGFP expression is restricted to the lateral membranes. Note that vinculin-EGFP expression is concentrated into foci in siblings but appears more diffuse in *flii^D110fs/D110fs^* zebrafish (magnifications shown in lower panel); each group, *n* = 5. Scale bars: projections, 25 μm; magnifications, 5 μm. (**B**) Plots of the relative pixel intensity along membranes from dotted boxed areas of **A**. Green and red dotted lines correspond to average minimum and maximum relative pixel intensities, respectively. Quantification of pixel intensity ratios is shown on the right. Unpaired *t* test; values represent means ± SEM; each group, *n* = 3. (**C**) Representative 3D views of 60 hpf *TgBAC(cdh2:cdh2-EGFP)* *flii^+/?^* sibling (left panels) and *flii^D110fs/D110fs^* cardiac ventricles (right panels). Magnifications show a clear punctate localization of cadherin2-EGFP in wild-type controls that is lacking in *flii^D110fs/D110fs^* embryos (*Z*-plane position color coded as indicated); each group *n* = 5. Scale bars: projections, 10 μm; magnifications, 10 μm. (**D**) Plots of the relative pixel intensity along membranes from dotted boxed areas of **C**. Green and red dotted lines correspond to average minimum and maximum relative pixel intensities, respectively. Quantification of pixel intensity ratios is shown on the right. Unpaired *t* test; values represent means ± SEM; *n* = 3 for each genotype.

To determine whether Flii is important for the formation and patterning of cardiomyocyte cell adhesion complexes, we directly visualized Vcl localization in vivo in *flii^D110fs/D110fs^* and *flii^+/?^* sibling larvae in the *Tg(myl7:vcla-EGFP)* transgenic background, expressing a Vcl-EGFP fusion protein in cardiomyocytes. At 60 hpf, Vcl-EGFP was predominantly localized at the lateral plasma membranes of ventricular cardiomyocytes in both homozygous mutant and sibling embryos. However, in *flii^+/?^* siblings, Vcl-EGFP was distinctly concentrated in foci (Figure 6A, left panel), whereas in *flii^D110fs/D110fs^* embryos, Vcl-EGFP was dispersed throughout the lateral cardiomyocyte membranes (Figure 6A, right panel). Quantification of relative pixel intensity of the profiles verified the presence of reduced Vcl-EGFP signal in foci in *flii^D110fs/D110fs^* embryos compared with *flii^+/?^* siblings (Figure 6B).

As described previously (31), Vcl-EGFP redistributes in wild-type zebrafish ventricular cardiomyocytes between 60 and 84 hpf, as it is no longer restricted to the lateral membranes but is also present in the apical and basal membranes of compact layer cardiomyocytes (Supplemental Figure 10, A–C). In *flii^D110fs/D110fs^* hearts, Vcl-EGFP appeared to undergo these same rearrangements (Supplemental Figure 10D). However, *flii^D110fs/D110fs^* hearts failed to form Vcl-EGFP foci at 84 hpf in the apical, basal, or lateral membrane compartments and instead exhibited evenly distributed Vcl-EGFP expression.

To determine whether Flii specifically regulates Vcl localization, or whether the loss of Flii causes a more general dysregulation of cell adhesion proteins, cadherin2-GFP localization was also evaluated in *flii^D110fs/D110fs^* hearts. Cadherin2 is a calcium-dependent transmembrane adhesion protein present in the intercalated discs of cardiomyocytes (32). Similar to the observed Vcl localization defects, mutant larvae displayed uniform distribution of cadherin2-GFP expression along the cell-cell junctions in contrast to the cadherin foci present in *flii^+/?^* siblings (Figure 6, C and D). These data indicate that Flii plays an important role in the establishment of functional cell adhesion complexes in the developing ventricular myocardium.

### Flii deficiency results in impaired Notch and Hippo signaling.

**Figure 7:**
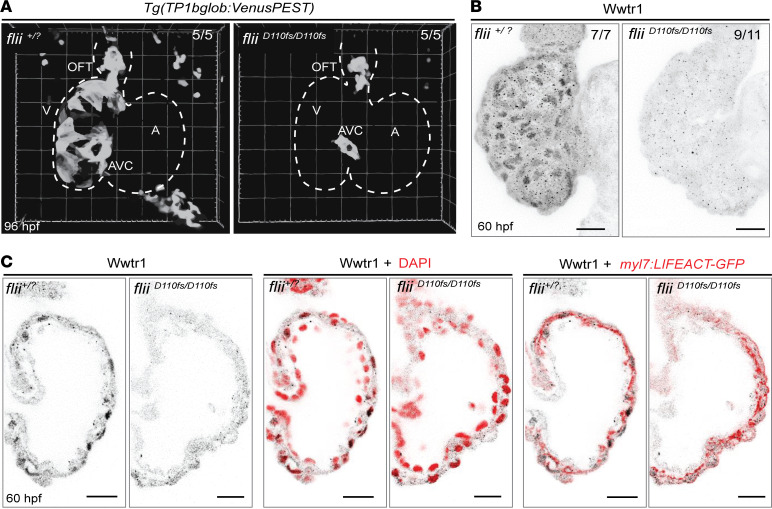
Aberrant activation of the Notch and Hippo signaling pathways in Flii-deficient ventricles. (**A**) Representative 3D volume renderings of *TP1bglob:VenusPEST* of *flii^+/?^* sibling and *flii^D110fs/D110fs^* hearts at 96 hpf. White dotted line outlines the heart. Note that there is no Notch reporter expression in the ventricle of *flii^D110fs/D110fs^* hearts, whereas there is expression in their AVC and OFT. Each group *n* = 5. V, ventricle; A, atrium; AVC, atrioventricular canal; OFT, outflow tract. (**B**) Representative maximum-intensity projections of wholemount *flii^+/?^* sibling and *flii^D110fs/D110fs^* hearts at 60 hpf stained for Wwtr1. *flii^+/?^* siblings *n* = 7, *flii^D110fs/D110fs^* *n* = 11. (**C**) Corresponding confocal sagittal sections of the wholemount ventricles shown in **B**. Nuclei are counterstained with DAPI, and cardiomyocyte F-actin myofibrils are marked with *myl7:*LIFEACT-GFP expression; scale bars, 20 μm.

A variety of signaling pathways have been linked to both DCM and cardiac trabeculation, including the Notch signaling pathway (33–36). During the process of trabeculation, Notch signaling becomes activated in a subset of compact layer cardiomyocytes in response to the cardiomyocytes that delaminate toward the ventricular lumen (35). Accordingly, impeding Notch signaling in cardiomyocytes has been reported to result in ventricular anomalies, including DCM (34). Hence, we used the established Notch signaling reporter line *Tg(TP1bglob:VenusPEST)* to investigate Notch activity in the myocardium of *flii^+/?^* sibling and *flii^D110fs/D110fs^* larvae (Figure 7A and Supplemental Videos 5 and 6). As previously described (35), at 96 hpf, a time point at which wild-type ventricles display substantial trabeculation, Notch signaling reporter expression was detected in wild-type compact layer cardiomyocytes as well as in the AVC and OFT. In contrast, although Notch reporter expression was present in the AVC and OFT of *flii^D110fs/D110fs^* larvae, it was strongly reduced in compact layer cardiomyocytes of the mutants.

The Hippo signaling pathway has also been identified as an important regulator of ventricular chamber morphogenesis (37) and the onset of DCM (38). In particular, the Hippo downstream effector Wwtr1/Taz has been shown to be important for ventricular wall maturation and Notch reporter expression in compact layer cardiomyocytes in a cell-autonomous way (37). To investigate whether Hippo signaling was also affected in the myocardium of *flii^D110fs/D110f^* hearts, the localization of Wwtr1/Taz in *flii^+/?^* siblings and mutants was assessed. Immunohistochemical analyses showed that Wwtr1/Taz nuclear localization was strongly reduced in the *flii^D110fs/D110f^* ventricular myocardium at 60 hpf (Figure 7, B and C). Taken together, these data show that Flii dysfunction not only affects structural components of the ventricular myocardium, including myofibrils and cell adhesion complexes, but it also results in the dysregulation of DCM-related signaling pathways during ventricular chamber morphogenesis.

## Discussion

This study describes 3 independent families with biallelic variants in *FLII* that lead to early-onset DCM. CRISPR/Cas9 genome editing in zebrafish and subsequent disease modeling verified that the discovered variants perturbed Flii function, which resulted in specific developmental defects of the heart and recapitulated key patient phenotypes in vivo. In addition, the *flii^D110fs^* mutant line that lacks a substantial part of the Flii protein, including the functional gelsolin domains, was examined to better understand the underlying disease-causing mechanisms. During ventricular chamber morphogenesis, Flii deficiency was found to lead to prominent structural defects in cardiomyocytes, affecting cell adhesion and myofibrillar architecture as well as causing severe trabeculation defects at the cellular level. In addition, Flii function was shown to be essential for the activation of 2 signaling pathways, Notch and Hippo, known to be involved in ventricular chamber morphogenesis. Together, these defects culminated in severely compromised cardiac wall morphogenesis, systolic heart failure, and larval lethality. In comparison with the *flii^D110fs^* mutants, genome-edited zebrafish containing patient-specific variants displayed a more subtle phenotype, with distinctive myofibrillar disorganization and concomitantly reduced ventricular contractility. Yet, the affected animals were able to survive past larval stages. Together, these data indicate that patient-specific alleles are indeed hypomorphic and that biallelic *FLII* variants in patients cause early-onset DCM through reduced FLII activity.

The gelsolin family member FLII was originally identified in *Drosophila* with hypomorphic alleles, which display an inability to fly that is caused by myofibrillar disorganization of the indirect flight muscles (39). Complete loss-of-function mutations, on the other hand, severely disrupt F-actin organization during cellularization and gastrulation (9, 10). In vitro studies have demonstrated the mechanistic importance of FLII in focal adhesions, where it associates with the actin cytoskeleton (6, 7, 29, 40). Taken together, these data point to a critical role for FLII in cell adhesion dynamics and associated cytoskeletal rearrangements. Nevertheless, the function of FLII in cardiomyocyte cell adhesion has not been previously studied to our knowledge. Our data provide evidence that Flii is present and functions in cardiomyocyte cell adhesion sites during ventricular chamber morphogenesis. Cell adhesion complexes, such as costameres and z-bodies, are crucial to maintain the structural and functional integrity of cardiomyocytes. Moreover, they play pivotal roles as mechanosensory units in signal transduction as well as in the adaptation to forces such as sarcomere contractions and hemodynamics (30, 41–43). Consequently, defects in cell adhesion proteins are known to cause contractile dysfunction and conduction abnormalities in various forms of inherited cardiomyopathies (44, 45).

During cardiac chamber morphogenesis, myofibrillar sarcomeric z-discs are initially attached to immature adhesion complexes (integrin-containing z-bodies) that allow reciprocal rearrangements between membrane contacts and sarcomeres (23). Myofibril maturation requires the subsequent recruitment of additional adhesion complex proteins such as Vcl and talin, leading to the formation of stronger and more stable connections between myofibrils and the plasma membrane (costameres) (23, 46). Zebrafish with impaired Flii function display mislocalization of Vcl and N-cadherin to intercellular adhesion sites in cardiomyocytes, indicating impaired formation of costameres and myofibril anchorage. Consequently, cardiomyocytes of *flii*-mutant hearts display disorganized myofibrils of reduced width. We speculate that cell adhesion instability and severely defective myofibril organization in *flii^D110fs^* mutants precede the architectural changes of cardiomyocytes that become spherical and protrude out of the epithelium-like myocardial layer at early larval stages. The absence of these ectopic cardiomyocytes in hypomorphic *flii^R1230C^* mutants can probably be attributed to residual Flii activity, and thus, more stable cell adhesion and enhanced integrity of cardiomyocytes.

The increasing hemodynamic load and tension during cardiac development results in biomechanical signaling and an adaptation of the heart chambers with the formation of ventricular trabeculae (47). Moreover, biomechanical signaling through actomyosin contractility is a driver of trabecular fate specification (35). Lack of trabeculation in mice and zebrafish results in early lethality as the heart is not able to support organismic growth (48, 49). To undergo trabeculation, cardiomyocytes are required to change their architecture by remodeling their cytoskeleton and cell adhesions while remaining attached to cardiomyocytes of the ventricular wall (32, 35). Interestingly, hearts of Vcl-knockout mice fail to form multilayered ventricular walls and exhibit reduced trabeculation (50). Taken together with our data, these observations indicate that stable and mature cell adhesion complexes along with proper myofibril organization are needed for the cardiac ventricle to undergo trabeculation. Accordingly, mislocalization of key cell adhesion proteins like Vcl and N-cadherin, caused by impaired Flii function, disrupts this essential step in the establishment of a competent ventricular wall. Of note, the active trabeculation process in zebrafish has been shown to increase the contractility of the myocardium between 96 and 120 hpf by increasing the surface area (22, 51). Consequently, reduced trabeculation, along with irregular organization of the myofibrils and z-discs in *flii*-mutant hearts, likely contributes to their progressive decline in ventricular contractility compared with wild-type animals.

In vertebrates, the Notch and Hippo signaling pathways are well-established regulators of multiple aspects of cardiovascular development, and the dysregulation of either pathway has been linked to the onset of DCM (33, 34, 38, 52). More specifically, the influence of tissue tension and biomechanical forces, together with components of both signaling pathways (and their crosstalk), is associated with the maturation of the ventricular wall (35). In line with these studies, we detected severely impaired Notch and Hippo signaling activity in the compact cardiac wall of *flii*-null mutants, which displayed trabeculation defects. The upstream mechanisms that initiate and control Notch and Hippo activity within the myocardium remain largely undefined. However, our data suggest that FLII might be a potential effector with an upstream regulatory role. In line with this idea, previous studies have reported the ability of FLII to function as a co-transcriptional regulator of nuclear receptors involved in cancer, inflammation, and wound healing, in addition to acting as a structural component of the cytoskeleton and possibly as a biomechanical sensor through cell adhesion complexes (53). However, further research is required to better understand the regulatory interactions underlying FLII function and the regulation of Notch and Hippo target genes during ventricular chamber morphogenesis.

Based on our findings, we propose an important role for FLII in cardiomyocyte cell adhesion and myofibril organization by regulating adhesion complex localization at an interface between myofibrils and their attachment to cardiomyocyte cell adhesion complexes. Impaired Flii function thus results in mislocalization of cell adhesion complex proteins including Vcl and cadherin, which, in cardiomyocytes, are crucial for proper myofibril organization and mechanosensation. Comparative sequence analysis of affected residues across FLII orthologs, together with computational analysis of FLII secondary and tertiary structures, revealed that amino acids mutated in patients were highly conserved and structurally important residues. We speculate that impaired FLII function in patients affects cell adhesion, myofibril organization, and mechanosensation, with consequences for the activation of downstream signaling pathways regulating ventricular chamber morphogenesis. Indeed, we and others found relocalization of cadherin2-containing adhesion complexes during trabeculation (25, 32), and previous reports link myofibril function, adhesion-regulating pathways, and mechanosensation to chamber maturation in fish and mammals (31, 50, 54, 55). This is further supported by our findings that Notch and Hippo signaling, 2 pathways important for ventricular chamber morphogenesis and linked to ventricular pathologies (35, 37), are aberrantly activated in Flii mutants. Together, our findings implicate perturbed myocardial cell adhesion and myofibril organization as the primary cause for the observed pathology in human patients; however, it will be necessary to test this hypothesis in patient-derived tissue.

In conclusion, this report describes biallelic variants in *FLII* as a novel genetic cause of pediatric CM. Using zebrafish disease modeling, we provide insights into the function of Flii during ventricular chamber morphogenesis that involves myocardial cell adhesion and myofibril organization. Further investigation of FLII and dissection of underlying disease-causing mechanisms, including preclinical compound screening in established zebrafish models, may reveal novel treatment opportunities that could help improve the prognosis of affected individuals.

## Methods

### Patients

#### Patient recruitment.

All affected probands were clinically evaluated by their treating clinical geneticist and pediatric cardiologist, including physical examination, 12-lead ECG, and transthoracic echocardiography. DCM was defined by the presence of LV dilation (LV end-diastolic dimension > 2 SD above the mean, scaled to body surface area) and systolic dysfunction (fractional shortening or LVEF > 2 SD below the mean for age) in the absence of abnormal loading conditions sufficient to cause global systolic impairment (56). After diagnosis of DCM, all first-degree family members were offered cardiac screening.

#### Whole-exome sequencing.

Genomic DNA (gDNA) was extracted from peripheral blood samples of the probands and their parents using standard procedures. Exons and flanking splice junctions were captured using the Agilent SureSelect Human All Exon kit. Sequencing was performed on an Illumina platform. Reads were aligned to the human reference genome GRCh37/hg19 using BWA (http://bio-bwa.sourceforge.net/), and variants were called using the GATK haplotype caller (https://www.broadinstitute.org/gatk/). Detected variants were annotated and filtered using Alissa Interpret software (Agilent). Priority was given to rare variants (minor allele frequency < 0.1% in public databases) that fit a recessive or de novo mode of inheritance. Sanger sequencing was used to verify all identified variants and test other family members.

### Zebrafish models

#### Zebrafish handling.

All zebrafish (*D*. *rerio*, strain: Tüb/AB) husbandry was conducted under standard conditions in accordance with institutional guidelines and national animal welfare legislation. Patient-specific zebrafish mutant lines *flii^re28^* (referred to in manuscript as *flii^S449fs^*) with a 7 bp deletion in exon 12 of the *flii* gene, leading to a frameshift mutation starting from amino acid position 449 and premature stop codon prior to the first gelsolin domain; *flii^re29^* (*flii^R1158W^*) harboring an Arg1158Trp substitution in exon 27 in the last gelsolin domain; and *flii^re30^* (*flii^R1230C^*) harboring an Arg1230Cys substitution in exon 30 in the last gelsolin domain were crossed into the *Tg(myl7:LIFEACT-GFP)s974* (22) background. Additionally, mutant line *flii^mi372^* (referred to in manuscript as *flii^D110fs^*), carrying a point mutation in exon 5 of the *flii* gene that leads to a frameshift mutation and a premature stop codon in the LRR domain (11), was crossed into the *Tg(myl7:LIFEACT-GFP)s974*, *Tg(myl7:vcla-EGFP)*bns24 (31), *Tg(TP1bglob:VenusPEST)*s940 (57), and *TgBAC*(*cdh2:cdh2-EGFP,crybb1:ECFP*)zf517 (58) backgrounds. The generation of newly constructed lines and corresponding genotyping strategies are described below.

Wild-type and transgenic adult zebrafish were maintained under standard laboratory conditions as described previously (59). Zebrafish embryos were maintained at 28°C in egg water (1 M HEPES-buffered [pH 7.2] E3 medium [34.8 g NaCl, 1.6 g KCl, 5.8 g CaCl_2_ × 2 H_2_O, 9.78 g MgCl_2_ × 6 H_2_O]). For high-speed imaging, and confocal microscopy, 0.2 mM 1-phenyl-2-thio-urea (Thermo Fisher Scientific) was added to the egg water at 24 hpf to prevent pigmentation.

#### Generation of CRISPR/Cas9 genome-edited zebrafish.

To introduce the patient-specific missense variants into the zebrafish genome, the CRISPR/Cas9 system was coinjected with a single-stranded DNA (ssDNA) oligo as described previously (19, 20). ssDNA oligos were purchased from Integrated DNA Technologies (IDT) as standard desalted DNA oligos. The Alt-R CRISPR/Cas9 System RNAs of IDT were used to generate single-guide RNAs (sgRNAs) with a specific target sequence. For the annealing condition, the Alt-R crispr RNA and Alt-R trans-activating crispr RNA were mixed in a 1:1 ratio in Duplex Buffer (IDT) and incubated at 95°C for 5 minutes, after which it was cooled down to room temperature. To assemble the ribonucleoprotein complex, 50 pmol of sgRNA was mixed with 4 ng Cas9 protein and incubated for 5 minutes at room temperature. Subsequently, 30 pmol ssDNA oligo was added to the mixture. Approximately 1.0 nL was microinjected into the yolk of 1-cell–stage embryos. Details of ssDNA oligos and target sequences can be found in Supplemental Table 2 and Supplemental Figure 3.

Injected embryos were raised to adulthood and outcrossed for germline transmission. To assess successful genome editing and germline transmission, roughly 20 F_1_ embryos per F_0_ founder were randomly selected and gDNA was extracted. A DNA fragment covering the CRISPR target site was amplified by PCR (see below, *Genotyping of zebrafish*) and sequenced by Sanger sequencing using primers listed in Supplemental Table 3. F_1_ siblings of genotyped embryos harboring patient-specific variants (*flii^S449fs/+^*, *flii^R1158W/+^*, or *flii*^R1230C/+^) were raised to adulthood and used to generate stable F_2_ mutants. Of note, the missense variant p.(L647V) variant identified in family 2 could not be modeled in zebrafish, because no sgRNA could be designed targeting the genomic region of interest.

#### Genotyping of zebrafish.

Adult zebrafish were anesthetized with 0.016% Tricaine, and a small portion of the caudal fin was excised. Larvae were anesthetized and harvested individually. Lysis was performed in either 80 μL 50 mM KOH or 1 mM proteinase K in Tris/EDTA buffer pH 7.4. gDNA from zebrafish larvae and adult tissue were isolated by incubating at 95°C until the tissue was completely dissolved. After lysis with KOH, 8 μL 1 M Tris-HCl pH 8 was added after incubation. Samples were centrifuged at 16,000*g* for 1 minute at room temperature, and 1 μL of sample was used for PCR. Genotyping of *flii^mi372^* (referred to as *flii^D110fs^*) animals was performed as previously described (11). For genotyping of patient-specific *flii* alleles, either a digestion or an allele-specific PCR was performed. See Supplemental Table 3 for corresponding method and primer sequences used. Amplification of DNA fragments was carried out in a total volume of 20 μL containing 2.0 μL of 10× FastStart Taq DNA Polymerase buffer, 1.6 μL of 2.5 mM dNTPs, 1.0 μL of 10 μM forward primer, 1.0 μL of 10 μM reverse primer, and 0.1 μL of FastStart Taq DNA Polymerase (Roche). PCR conditions were as follows: initial denaturation at 95°C for 5 minutes; followed by 10 cycles of denaturation at 95°C for 30 seconds, annealing at 65°C to 55°C for 30 seconds by decreasing 0.5°C steps cycle-wise, and extension at 72°C for 45 seconds; followed by 25 cycles of denaturation at 93°C for 30 seconds, annealing at 58°C for 30 seconds, and extension at 72°C for 45 seconds; followed by a final extension step at 72°C for 3 minutes.

For allele-specific PCR, 1.0 μL of 10 μM allele-specific primer was also added to the reaction mixture (in a 1:1:1 ratio with general primers). For digestion, 5 μL of PCR product was used in a total volume of 20 μL. PCR products were loaded onto a 2.2% agarose gel (Sigma-Aldrich) in 1× Tris-acetate-EDTA buffer (40 mM Tris, 20 mM acetic acid, 1 mM EDTA) (Sigma-Aldrich).

### Cardiac contractility analysis

Heart rate and ventricular contractility parameters were obtained using bright-field, in vivo, high-speed imaging (20, 21). Briefly, larvae were anesthetized in 0.02% Tricaine and mounted in a ventral position in 0.25% (*w/v*) agarose (MilliporeSigma) on glass-bottom Petri dishes. Videos were acquired at 150 frames/s for a total length of 10 seconds using a C9300-221 high-speed charge-coupled device camera (Hamamatsu Photonics) mounted on a DM IRBE inverted microscope (Leica Microsystems). Following video acquisition, larvae were gently removed for independent post hoc genotyping. Recordings were analyzed using Fiji ImageJ software (60). Cardiac contractility parameters were derived by manually outlining the perimeter of the ventricle at end-diastole and end-systole. EDV and ESV were calculated by (1/6) × π × major axis × (minor axis^2^). Stroke volume was calculated by EDV – ESV. EF was derived from stroke volume/EDV. Ventricular area at end diastole and end systole was calculated by (0.5 × major axis) × (0.5 × minor axis) × π. The FAC was calculated by: (area diastole − area systole)/area diastole.

### Blood flow analysis

Blood flow was recorded by imaging the dorsal aorta between the eighth and tenth segmental vessels using a spinning disk microscope (Zeiss Observer Z.1). Larvae were anesthetized in 0.02% Tricaine and mounted in 2% low-melting-point agarose on glass-bottomed Petri dishes. Videos were acquired at 400 frames/s for a total length of 2 seconds. Kymographs were generated from blood flow recordings in Fiji and were used to determine BFV. Blood cell velocity and acceleration were measured by single-cell tracking using a particle tracking software Tracker (http://physlets.org/tracker/).

### Immunofluorescence staining

Adult zebrafish hearts were dissected and prepared for embedding in OCT tissue-freezing medium. Sections were stained using standard immunofluorescence conditions (anti-FliI, 1:500, Hiromi Hirata, Ayomama Gakuin University, Sagamihara, Japan; Alexa Fluor, anti-rabbit 568, 1:200, Thermo Fisher Scientific). Wwtr1 staining using the Wwtr1 antibody (Cell Signaling Technology, D24E4) was performed as previously described (37).

### Confocal microscopy

Confocal imaging was performed using a confocal laser scanning microscope LSM 700 (Carl Zeiss) or SP5 Intravital (Leica Microsystems). Tricaine-anesthetized embryos were mounted in 2% low-melting-point agarose in egg water on glass-bottomed Petri dishes. Hearts were imaged immediately after cardiac arrest. Wholemount hearts were manually isolated from fixed animals and embedded in 1% agarose. 3D renderings of wild-type and mutant lines in the *Tg(myl7:LIFEACT-GFP)* background were generated with Imaris Bitplane (Oxford Instruments). Imaging of *Tg(TP1bglob:VenusPEST*)s940 and *TgBAC(cdh2:cdh2-EGFP,crybb1:ECFP*)zf517 was performed as previously described (35, 61).

### Confocal image analysis

Confocal fluorescence image analysis was carried out using Fiji ImageJ software. Cardiac trabeculation was quantified by counting cardiomyocytes protruding out of the single-layered, compact myocardium in confocal single planes of the ventricular outer curvature. To quantify fluorescence intensity, the profile plotting tool was used.

### TEM

TEM of zebrafish embryos was largely performed as described previously (62). Larvae were collected and immediately fixed in ice-cold 1%–2% PFA, 2% glutaraldehyde in 0.1 M sodium cacodylate buffer (pH 7.4) for 30 minutes on ice, then stored at 4°C. Samples were washed in 0.1 M sodium cacodylate buffer and postfixed in 2% (*w/v*) OsO_4_. Samples were dehydrated with a graded series of washes in acetone, transferred to acetone/Epon solutions, and eventually embedded in Epon. Ultrathin sections (approximately 60 nm) obtained with a Reichert-Jung Ultracut E microtome were collected on copper slot grids. Sections were contrasted with uranyl acetate and lead citrate and examined with either a Philips CM10 transmission electron microscope operated at an accelerating voltage of 80 kV or a Jeol JEM-1400 Plus transmission electron microscope operated at an accelerating voltage of 120 kV. The researcher performing the imaging analysis did not know the genotype of the samples.

### Reverse transcription PCR

For reverse transcription PCR, RNA was isolated from zebrafish embryos (*n* ≥ 25) at the indicated developmental stages using RNeasy Kit (QIAGEN). First strand synthesis was performed using Superscript II (Invitrogen) following the manufacturer’s instructions. Reverse transcription PCR was performed following standard protocols (primers for *flii*, 5′-AATGCCAGGTCTTCAAATCC-3′ and 5′-TTTCATCTGGTCCTTCTGCT-3′, and *18S rRNA*, 5′-GTTGATTAAGTCCCTGCCCT-3′ and 5′-TTTACTTCCTCTAAACGACCGA-3′).

### Transcriptomic data reanalysis

To investigate *flii* expression in the zebrafish heart in single-cell resolution, we obtained raw count matrices of scRNA-Seq from zebrafish hearts (Gene Expression Omnibus GSE106121) (63) and reanalyzed the data as described (64). Data visualization was performed using scanpy dot plot function including marker gene expression for individual cardiac cell types as control. Analysis of *FLII* expression in the human heart in single-nucleus resolution is based on the public data from the Human Heart Cell Atlas, Global heart data set, youngest healthy age group (40–45 years).

### Quantitative PCR of flii in zebrafish larvae

For quantitative PCR (qPCR), RNA was isolated from zebrafish larvae of known genotype at 5 dpf (*n* = 20), using TRIzol Reagent (Thermo Fisher Scientific), followed by cDNA synthesis using the iScript cDNA Synthesis Kit (Bio-Rad). qPCR was performed in triplicate using iTaq Universal SYBR Green Supermix on a CFX96RTS thermal cycler, in triplicate (Bio-Rad). The relative gene expression levels were determined using the ΔΔct method. For the qPCR analysis the following primer pairs were used: for *flii*, 5′- AAGGGCTATGCAGGTGTGG-3′ and 5′- CCAGCTCAGTGAGGAAATGG-3′, and β-actin, 5′-TCCTGGGTATGGAATCTTGC-3′ and 5′-GCACTGTGTTGGCATACAGG-3′.

### In silico protein 3D modeling

PDB files of human and zebrafish flii protein were retrieved from Alphafold-2 with accession numbers Q13045 and F8WK50, respectively. Visualization of 3D structure and in silico mutagenesis was performed using PyMol (Schrödinger Inc.). The Find function was used to identify potential hydrogen bonds.

### Statistics

Statistical analyses were performed using GraphPad Prism v.6 software and consisted of 2-tailed *t* test or 1-way ANOVA followed by the Tukey or Holm-Šídák post hoc test, as stated in figure legends. Results are expressed as mean ± SEM, unless otherwise indicated. For our study, a value of *P* < 0.05 was considered statistically significant. Whenever possible, masking was performed in data collection and analysis.

### Study approval

This study does not fall within the scope of the Medical Research Involving Human Subjects Act (WMO) and therefore does not need to be reviewed and authorized by the institutional review board. Written informed consent for genetic testing and publication of anonymized data were obtained from the legal guardians of the affected probands prior to inclusion in this study. For animal studies, we have complied with all relevant ethical regulations in accordance with and approved by institutional guidelines and national animal welfare legislation.

### Data availability

All individual data points included in the figures and statistical analyses are available in the Supporting Data Values. Additional supporting data are available on reasonable request from the corresponding authors. Our ethics committee does not allow sharing of human genotype information in the public domain.

## Author contributions

CWBR and FH participated in experimental design; performed research; collected, analyzed, and interpreted data; performed statistical analysis; and drafted and revised the manuscript. HI, MPH, J Pestel, LK, JKHL, HCVDL, RW, and J Piesker performed research and interpreted data. ZNAH, AA, MD, LMVDB, and MAVS interpreted and provided clinical data. CWBR, FH, MPH, J Pestel, LK, JKHL, HCVDL, RW, J Piesker, ZNAH, AA, MD, LMVDB, MAVS, FT, JB, TJVH, DYRS, JMAV, and SR contributed to the revision of the manuscript and read and approved the final version. TJVH, DYRS, JMAV, and SR supervised the study. CWBR and FH are co–first authors. The order of co–first authors was decided following transparent discussion and was based on the efforts and contributions to the manuscript.

## Supplementary Material

Supplemental data

Supplemental video 1

Supplemental video 2

Supplemental video 3

Supplemental video 4

Supplemental video 5

Supplemental video 6

Supporting data values

## Figures and Tables

**Table 1:**
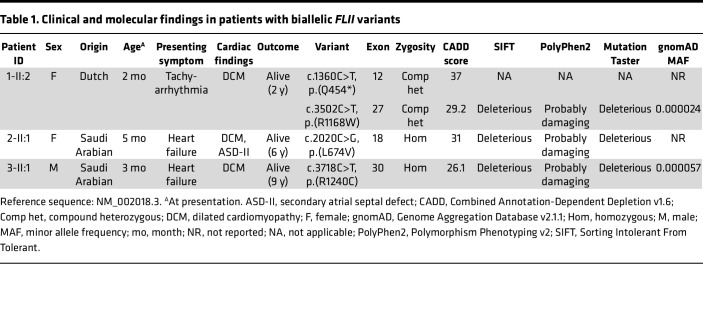
Clinical and molecular findings in patients with biallelic *FLII* variants

## Version 1 08/10/2023

In-Press Preview

## Version 2 09/08/2023

Electronic publication
