# Supplementary material for: Biallelic variants in FLII cause pediatric cardiomyopathy by disrupting cardiomyocyte cell adhesion and myofibril organization
Source: JCI Insight. 2023 Sep 8;8(17):e168247. doi: 10.1172/jci.insight.168247 (PMC10544232; doi:10.1172/jci.insight.168247)
Supplement: Supplemental data [file jciinsight-8-168247-s021.pdf]

SUPPLEMENTAL MATERIALS

Bi-allelic variants in *FLII* cause pediatric cardiomyopathy by disrupting cardiomyocyte cell adhesion and myofibril organization

Claudine W.B. Ruijmbeek, Filomena Housley, Hafiza Idrees, Michael P. Housley, Jenny Pestel, Leonie Keller, Jason Kuan Han Lai, Herma C. van der Linde, Rob Willemsen, Janett Piesker, Zuhair N. Al-Hassnan, Abdulrahman Almesned, Michiel Dalinghaus, Lisa M. van den Bersselaar, Marjon A. van Slegtenhorst, Federico Tessadori, Jeroen Bakkers, Tjakko J. van Ham, Didier Y. R. Stainier, Judith M.A. Verhagen, and Sven Reischauer

Supplemental Figures and Figure Legends 1 – 10

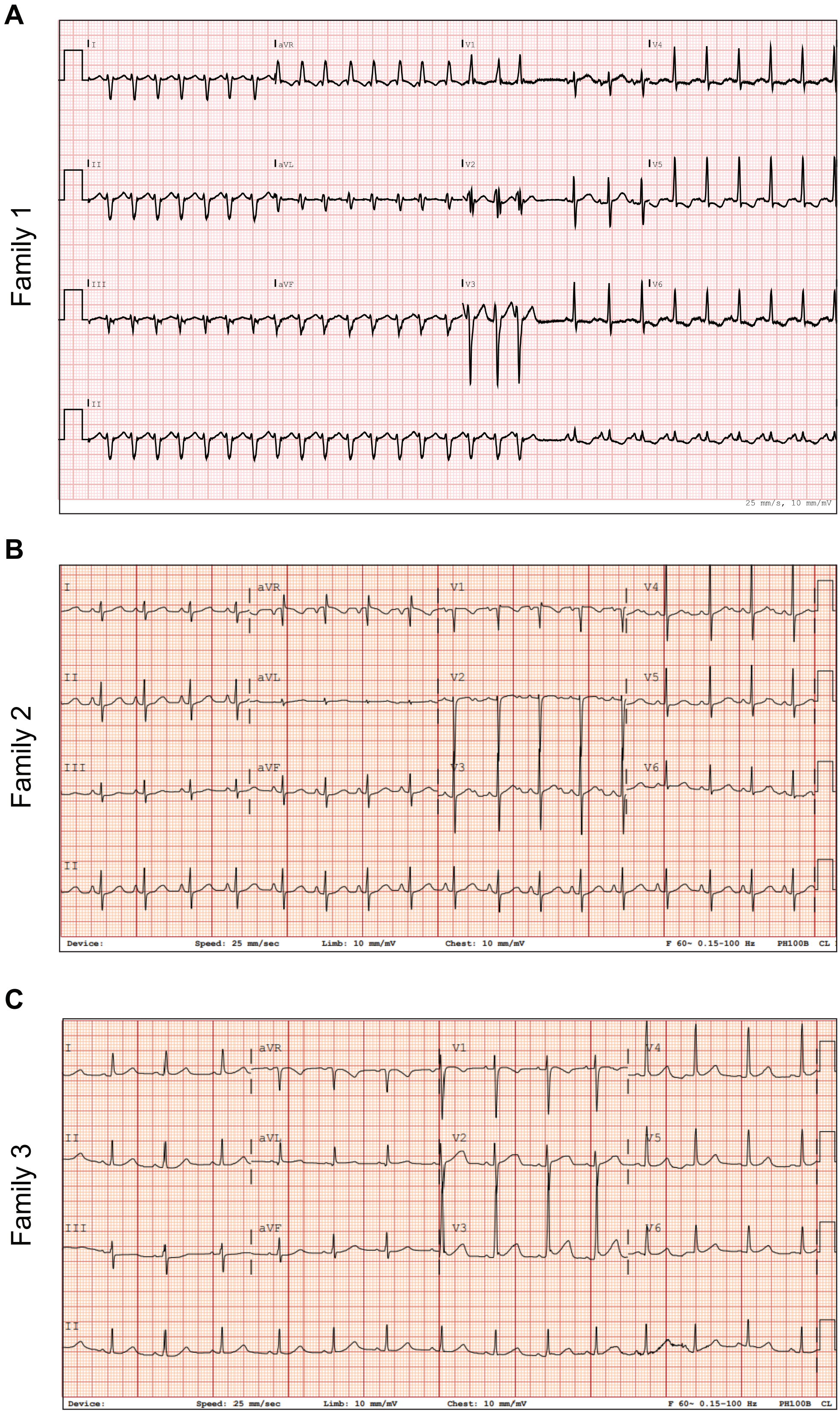

Supplemental Figure 1. Electrocardiograms of pediatric cardiomyopathy patients at presentation. **A**, ECG of patient 1-II-2 showing ventricular tachycardia converting to sinus rhythm. **B**, ECG of patient 2-II-1. **C**, ECG of patient 3-II-1.

Supplemental Figure 2

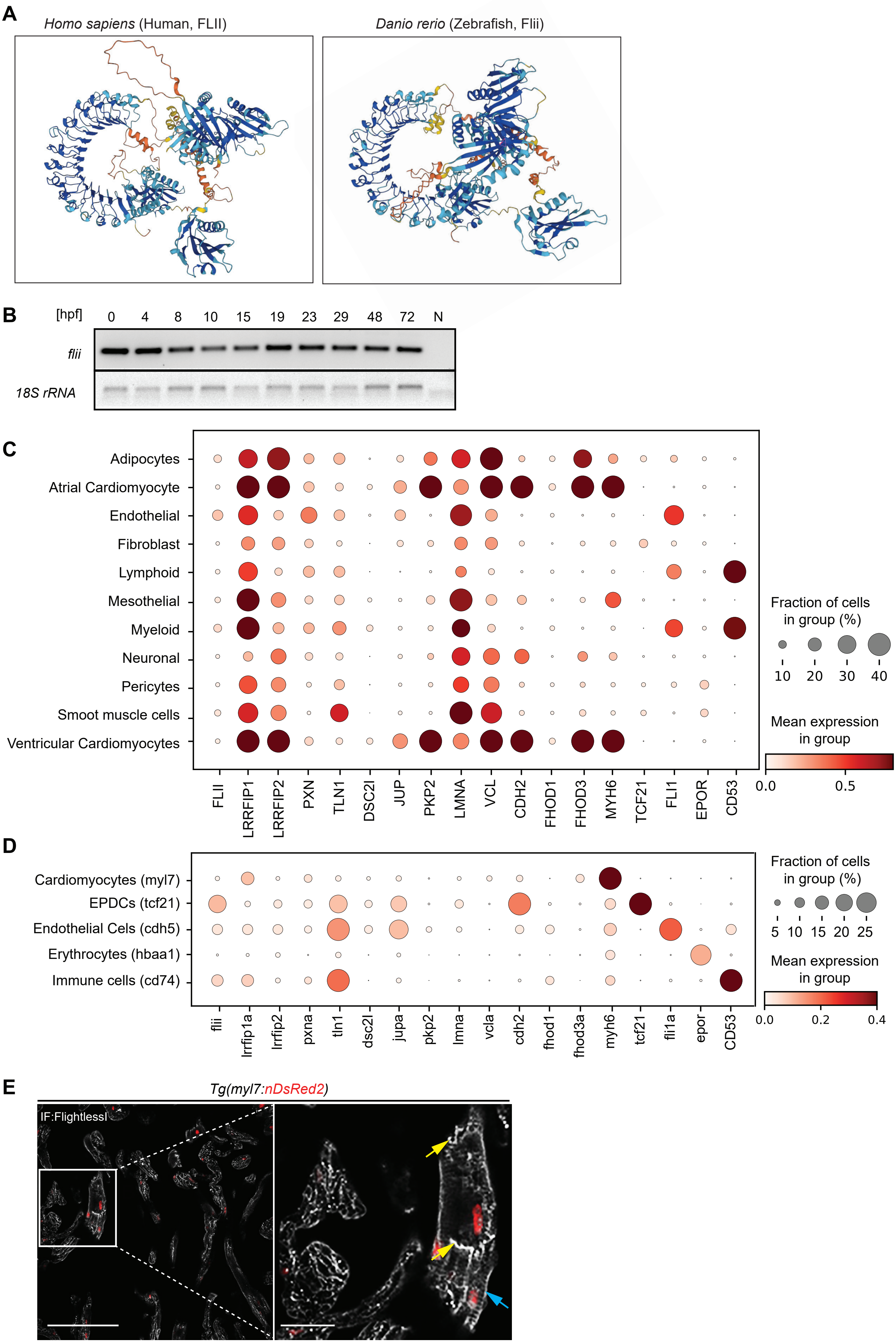

Supplemental Figure 2. Flii is expressed in cardiomyocytes. A, Tertiary structure of the human and zebrafish flightless-1 proteins as predicted by Alphafold-2 with accession numbers: Q13045 and F8WK50, respectively. **B**, *flii* temporal expression during zebrafish development as assessed by RT-PCR. *flii* is expressed at all stages examined. 18S rRNA serves as loading control; N, negative control. **C-D**, Gene expression data obtained from scRNA-seq data of human (**C**) and zebrafish (**D**) heart tissue showing the expression of selected genes in various cardiac cell types. **E**, Immunofluorescent staining for Flii in sections of adult *Tg(myl7:*nDsRed2*)* zebrafish ventricular myocardium. Yellow arrows, intercalated disc; blue arrow, costameres; red staining, nuclei; IF, immunofluorescence; scale bars: left 100 μm, right 25 μm.

Supplemental Figure 3

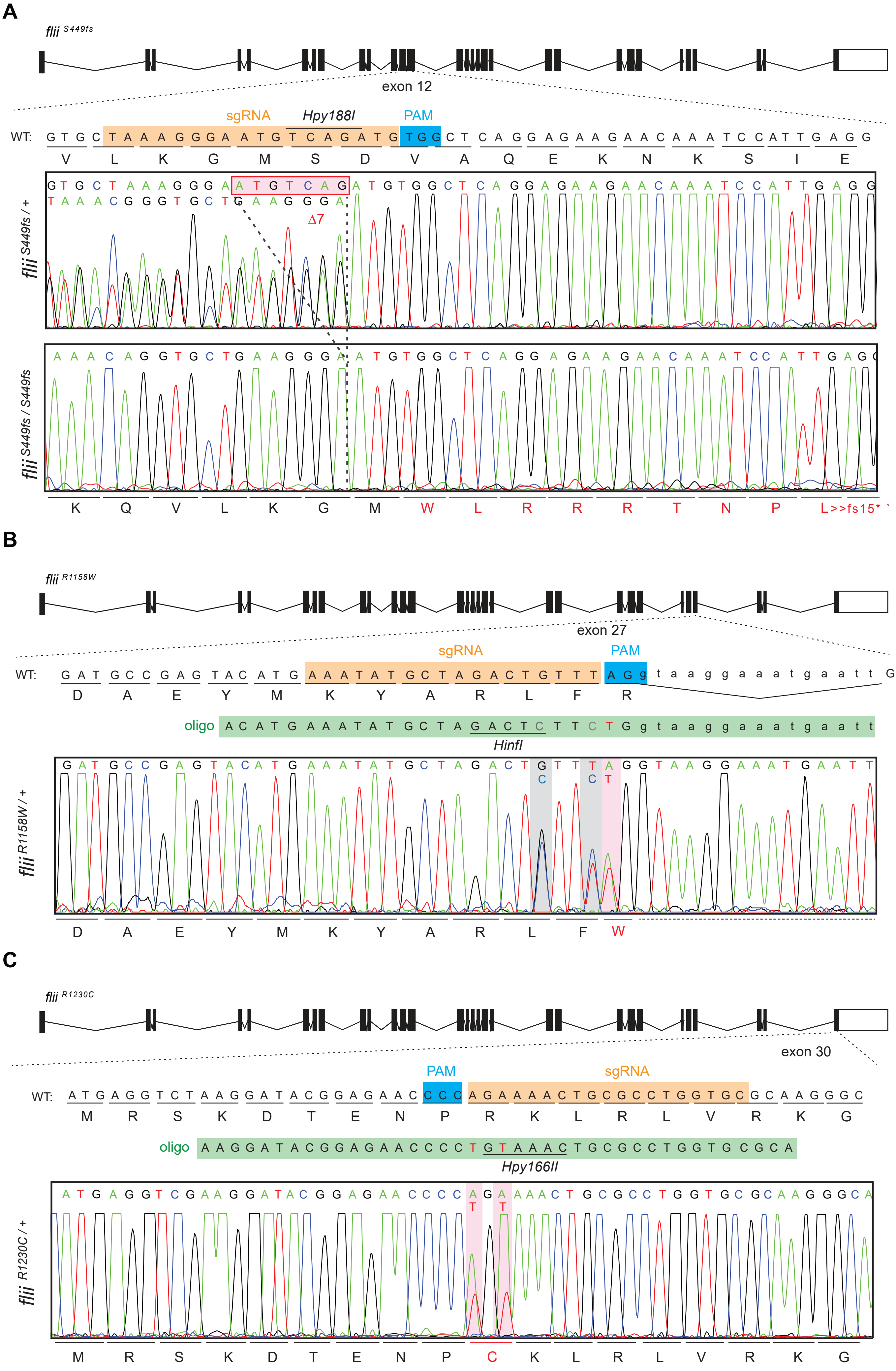

Supplemental Figure 3. CRISPR/Cas9-mediated precision genome editing strategy in detail. A, Strategy to generate the *flii*^{449fs} frameshift variant mimicking the stop variant p.(Q454*) identified in family 1. The top panel shows a schematic representation of the zebrafish *flii* gene with its total of 30 exons. The 20-nucleotide genomic target sequence in exon 12 is highlighted in orange, and the adjacent PAM site is shown in blue. The *Hpy188I* restriction site used for genotyping is underlined. Codons and corresponding amino acids are also indicated. The lower panel shows the Sanger sequencing trace of a heterozygous *flii*$^{449fs/+}$ larva (sequence is shown reverse-complement as the reverse primer was used for sequencing), displaying a frameshift mutation caused by a 7-bp deletion at the predicted target site, indicated by the double base calls. For clarity reasons, a sequence trace of a homozygous *flii*$^{499fs/449fs}$ larva is also depicted. The frameshift terminates 15 amino acids after the first amino acid change. **B**, The top panel shows the strategy used to generate the *flii*^{R1158W} missense variant mimicking p.(R1168W) identified in family 1. The target sequence in exon 27 is highlighted in orange, PAM is shown in blue, and the co-injected 40 bp oligo containing the R1158W missense variant is highlighted in green. The novel *HinfI* restriction site is underlined. Lowercase letters indicate intronic sequences. The lower panel displays the Sanger sequencing trace of a heterozygous *flii*$^{R1158W/+}$ zebrafish. Synonymous nucleotide changes are highlighted in grey, and the missense variant is highlighted in red. **C**, The top panel shows the strategy used to generate the *flii*^{R1230C} missense variant p.(R1240C) identified in family 3. As in **B**, the target sequence in exon 30 is highlighted in orange, the PAM is in blue, and the co-injected 40-bp oligo containing the R1230C missense variant is in green. The novel *Hpy16II* restriction site is underlined. The lower panel displays the Sanger sequencing trace of a heterozygous *flii*$^{R1230C/+}$ zebrafish in which the missense variant is highlighted in red. PAM, protospacer a djacent motif; oligo, oligonucleotide.

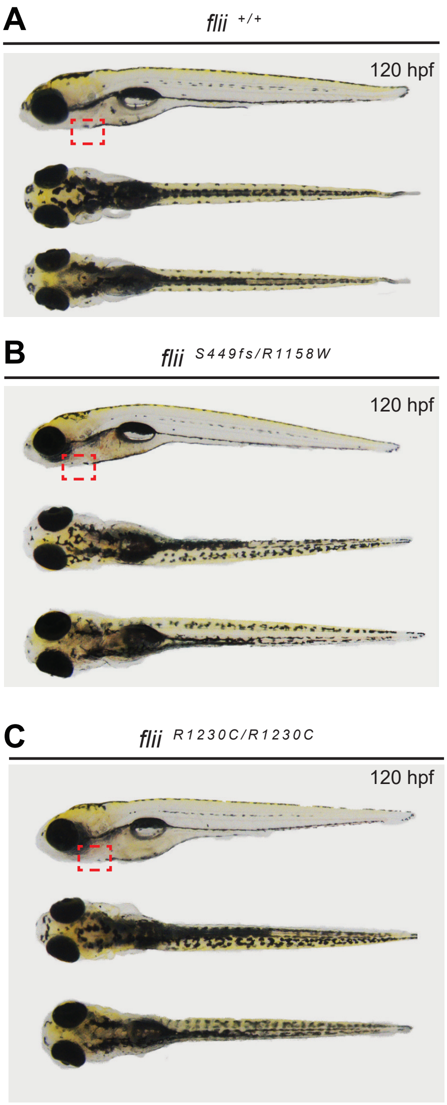

Supplemental Figure 4. Upon gross examination mutant larvae harboring patient-specific variants are indistinguishable from wild-types. A-C, Representative images of zebrafish larvae seen from a lateral (top), dorsal (middle), and ventral (bottom) views. **A**, Wild-type $flii^{+/+}$ 120 hpf larvae. Red-dashed square depicts heart region. **B**, Compound heterozygous $flii^{S449fs/R1158}$; **C**, $flii^{R1230C/R1230C}$. At least *n*=20 for every genotype were morphologically screened.

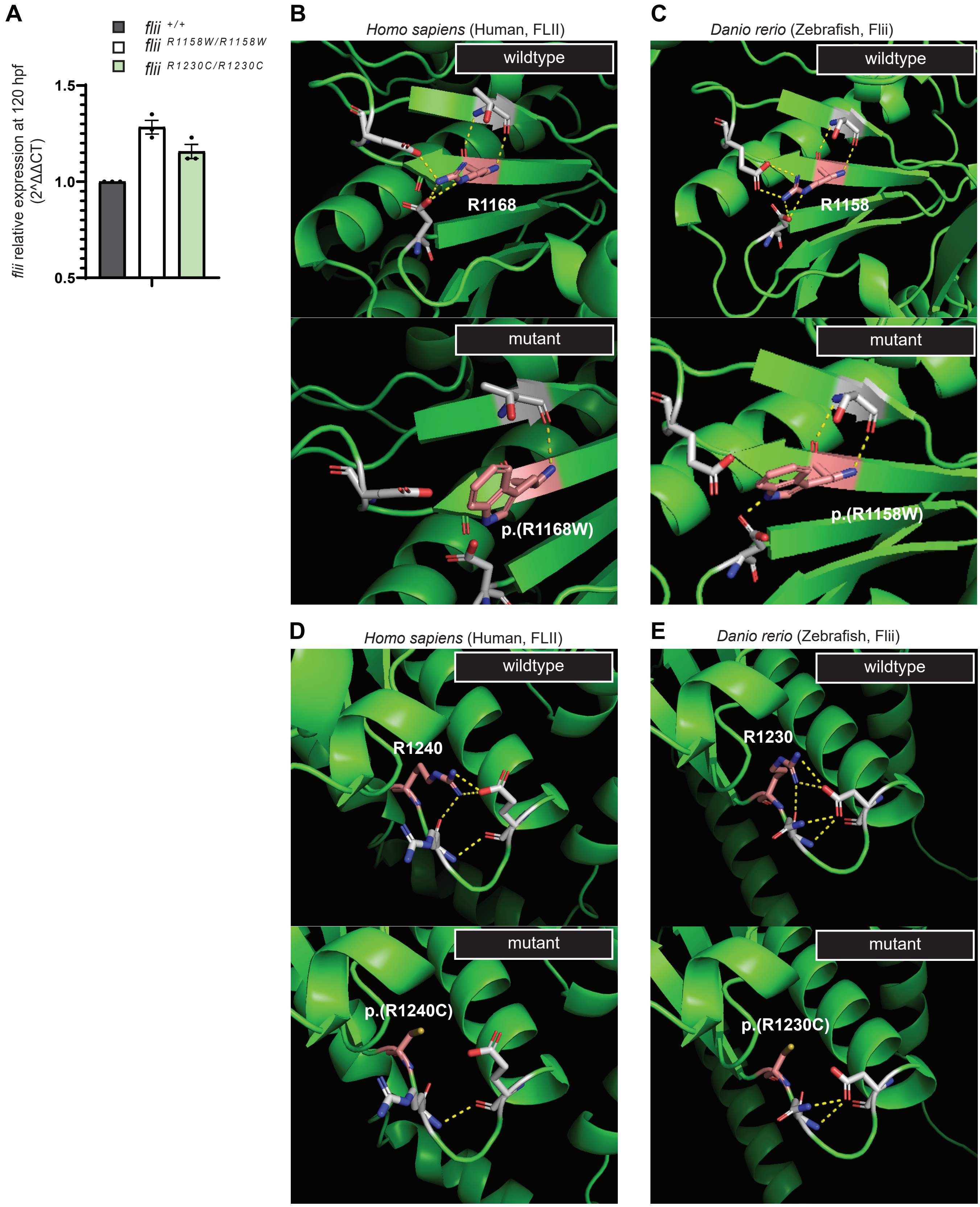

Supplemental Figure 5. Consequences of *flii* variants. A, Expression of *flii* mRNA levels in 120 hpf *flii*$^{+/+}$, *flii*$^{R1158W/R1158}$ and *flii*$^{R1230C/R1230C}$ zebrafish larvae normalized to β-actin. Note, that there is no sign of mRNA degradation. To investigate the influence of the R1158W missense variant, homozygous *flii*$^{R1158W/R1158W}$ larvae were used. For every genotype, *n*=3 technical replicates were evaluated. **B-E**, Computational 3D modeling of human (**B** and **D**) and zebrafish (**C** and **E**) wild-type and mutant flightless-1 proteins in PyMol, using PDB files derived from Alphafold-2, with accession numbers Q13045 and F8WK50, respectively. Carbons of native and mutagenized amino acid of interest (R1168 in humans in (**B**), corresponding to R1158 in zebrafish in (**C**), and R1240 in humans in (**D**), corresponding to R1230 in zebrafish in (**E**)) are shown in stick and colored in red. Interacting amino acids are also shown in stick, and carbon atoms are colored in grey. Hydrogen bonds are indicated as dotted lines.

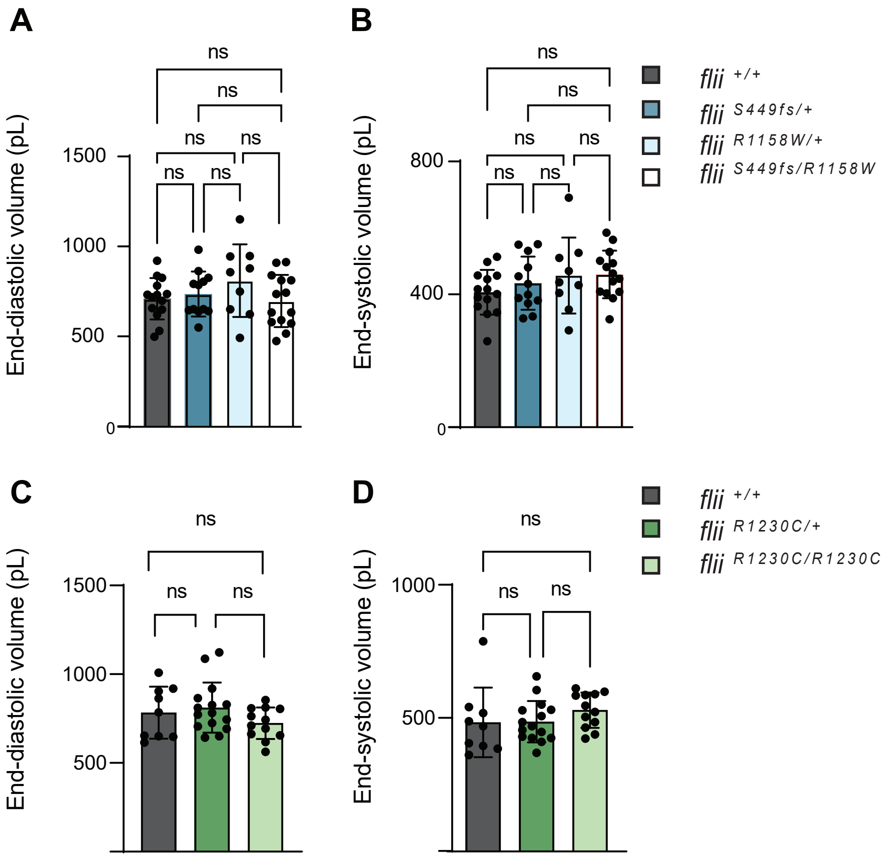

Supplemental Figure 6. Ventricular dimensions of mutant larvae harboring patient-specific variants derived from high-speed imaging movies. A-B, End-diastolic volume (**A**) and end-systolic volume at 120 hpf (**B**) for $flii^{+/+}$, $flii^{S449fs/+}$, $flii^{R1158W/+}$, and $flii^{S449fs/R1158W}$. $flii^{+/+}$ *n* = 14; $flii^{S449fs/+}$ *n* = 12; $flii^{R1158W/+}$ *n* = 9; $flii^{S449fs/R1158W}$ *n* = 14. **C-D**, End-diastolic volume (**C**) and end-systolic volume (**D**) for $flii^{+/+}$, $flii^{R1230C/+}$ and $flii^{R1230C/R1230C}$. $flii^{+/+}$ *n* = 9; $flii^{R1230/+}$ *n* = 15; $flii^{R1230C/R1230C}$ *n* = 12. Statistics: mean ± SD; One-way ANOVA coupled with Tukey's multiple comparison test was used to test for significance.

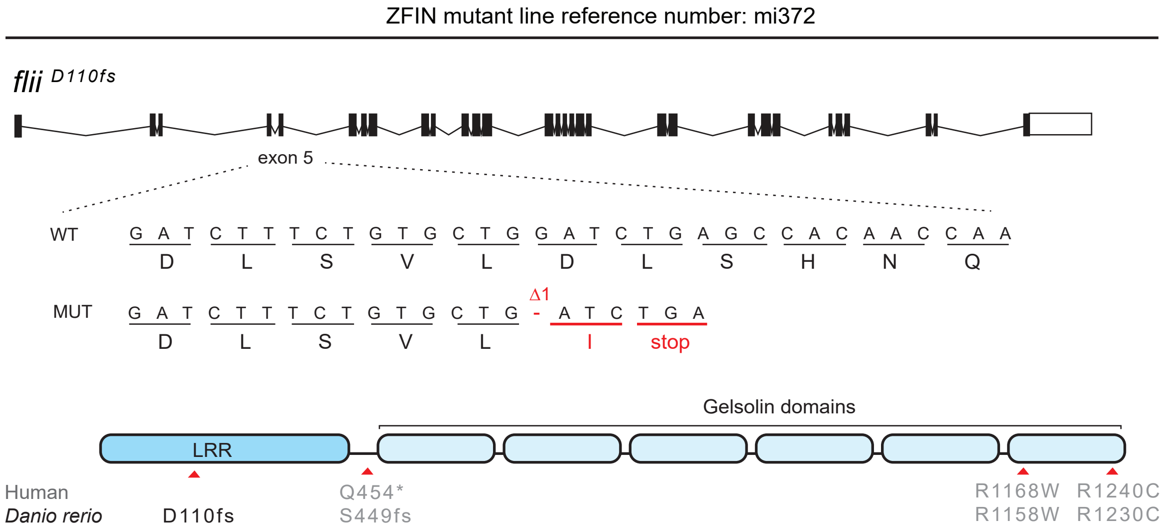

Supplemental Figure 7. Flii-deficient mutant zebrafish allele. Schematic representation of the *fliimi372* allele (1) (referred to in manuscript as $flii^{D110fs}$). A single base deletion in exon 5 leads to a frame shift at amino acid position 110, resulting in a premature stop in the LRR domain.

Supplemental Figure 8

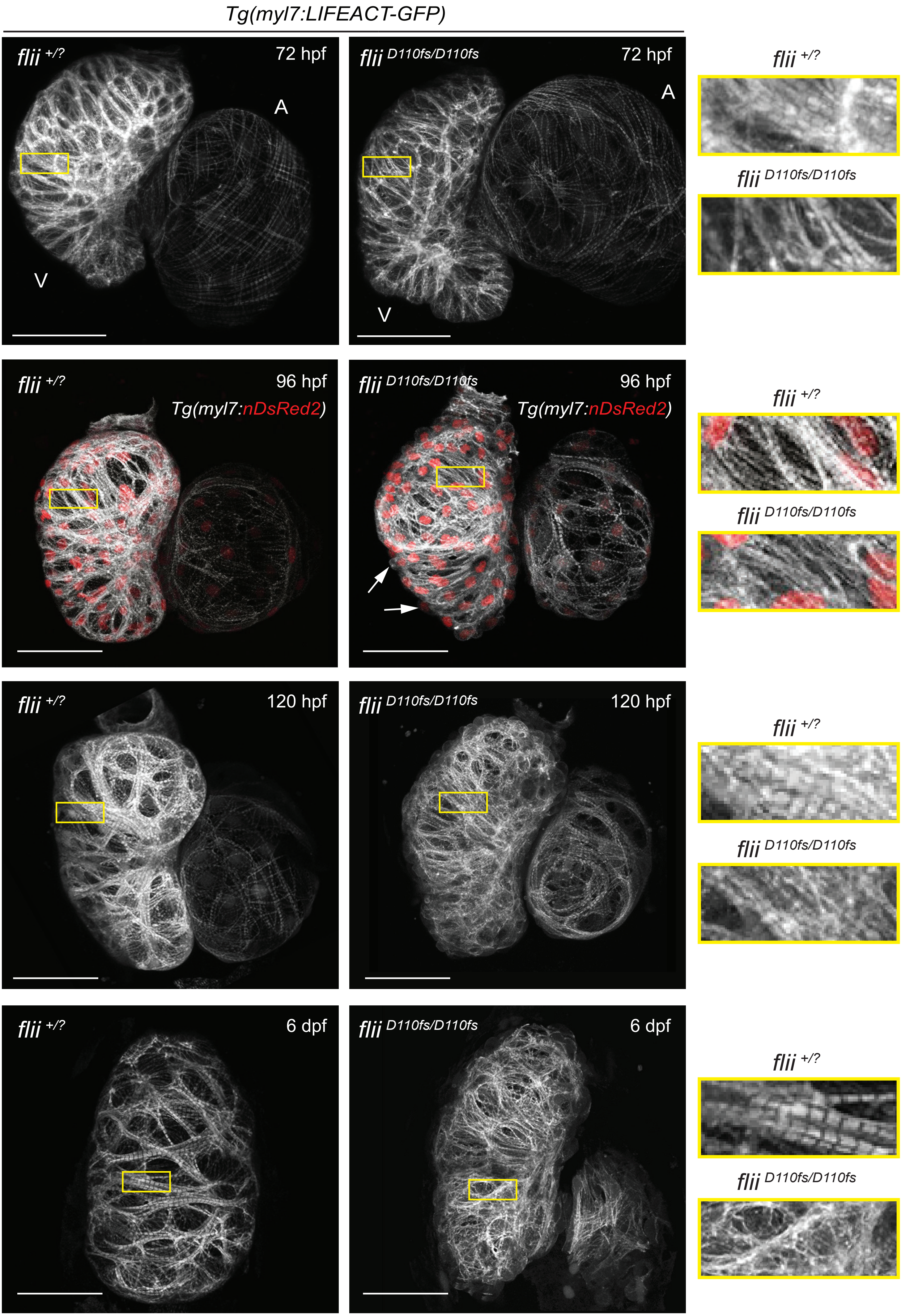

Supplemental Figure 8. Flii-deficient zebrafish display cardiomyocyte cytoskeletal defects as early as 72 hpf. 3D confocal projections of *Tg(myl7:LIFEACT-GFP)* hearts at 72 hpf - 6 dpf. Left panels show wild-type and heterozygous siblings referred as *flii*$^{+/?}$ siblings and right panels show *flii*$^{D110fs/D110fs}$ animals. 96 hpf hearts express *Tg(myl7:*nDsRed2*)* to visualize cardiomyocyte nuclei. Magnifications of representative areas of sibling and mutant myofibrils are provided in adjacent yellow boxes. White arrows point to individual protruding cardiomyocytes that have lost their epithelial-like cardiomyocyte shape. Note that ventricular myofibrils appear thinner and less organized in *flii*$^{D110fs/D110fs}$ hearts as early as 72 hpf. No cardiac phenotype could be detected in heterozygous *flii* larvae and therefore only one representative image is shown. *flii*$^{+/?}$ siblings *n* = 8, *flii*$^{D110fs/D110fs}$ *n* = 5; scale bars: 50 µm. V, ventricle; A, atrium.

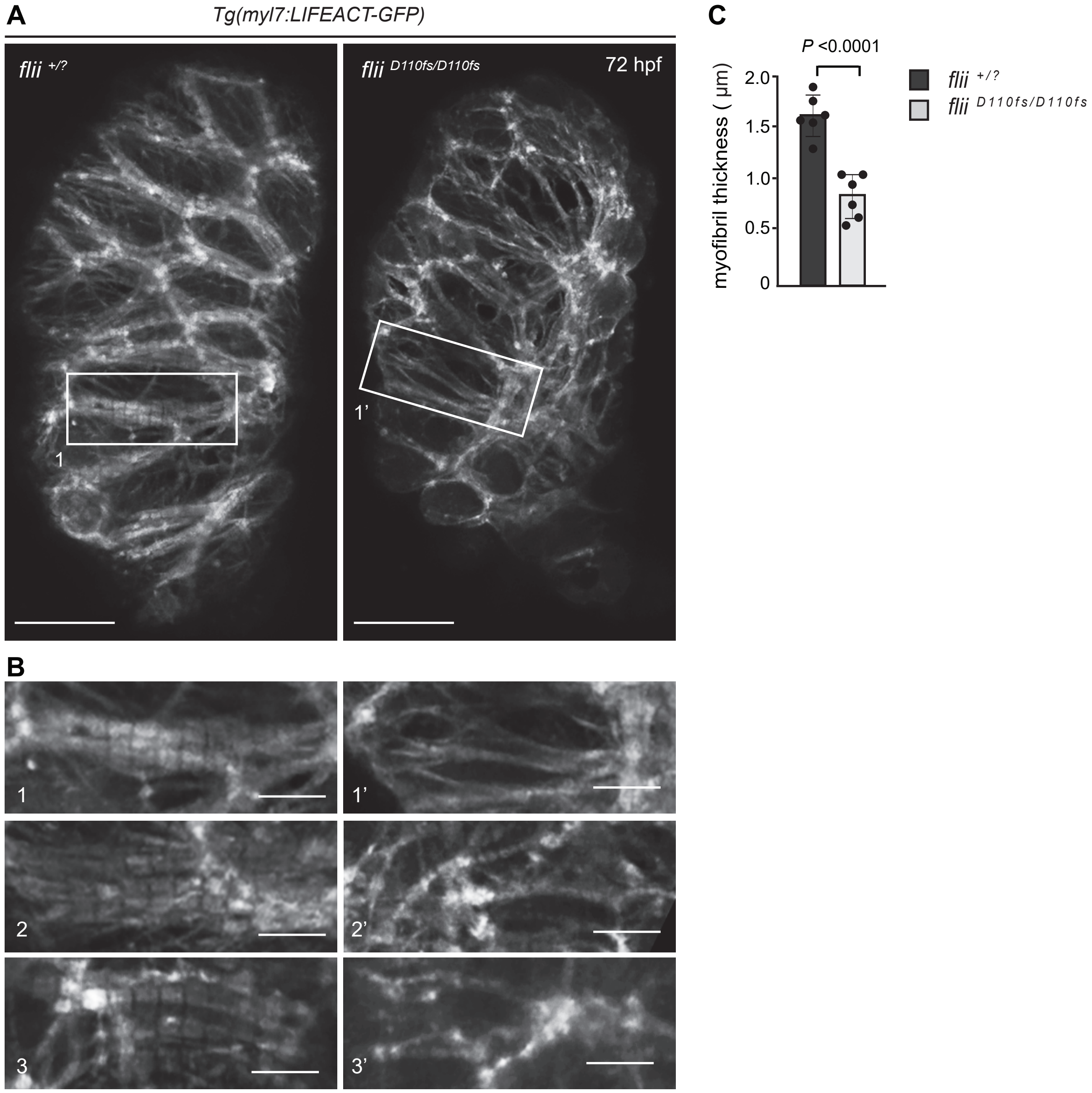

Supplemental Figure 9. Myofibrils fail to bundle and mature in Flii-deficient zebrafish. A, 3D confocal projections showing ventricular surfaces of 72 hpf *Tg(myl7:LIFEACT-GFP) flii*$^{+/?}$ siblings (left panel) and *flii*$^{D110fs/D110fs}$ larvae (right panel). Scale bars: 20 µm, B, Magnifications of representative areas of sibling and mutant myofibrils of white boxes in (**A**). Scale bars: 5 µm. **C**, Analysis of myofibril thickness (µm) from presented areas shown in (**B**). Unpaired t-test, values represent means ± SEM; *flii*$^{+/?}$ siblings *n* = 6, *flii*$^{D110fs/D110fs}$ *n* = 6.

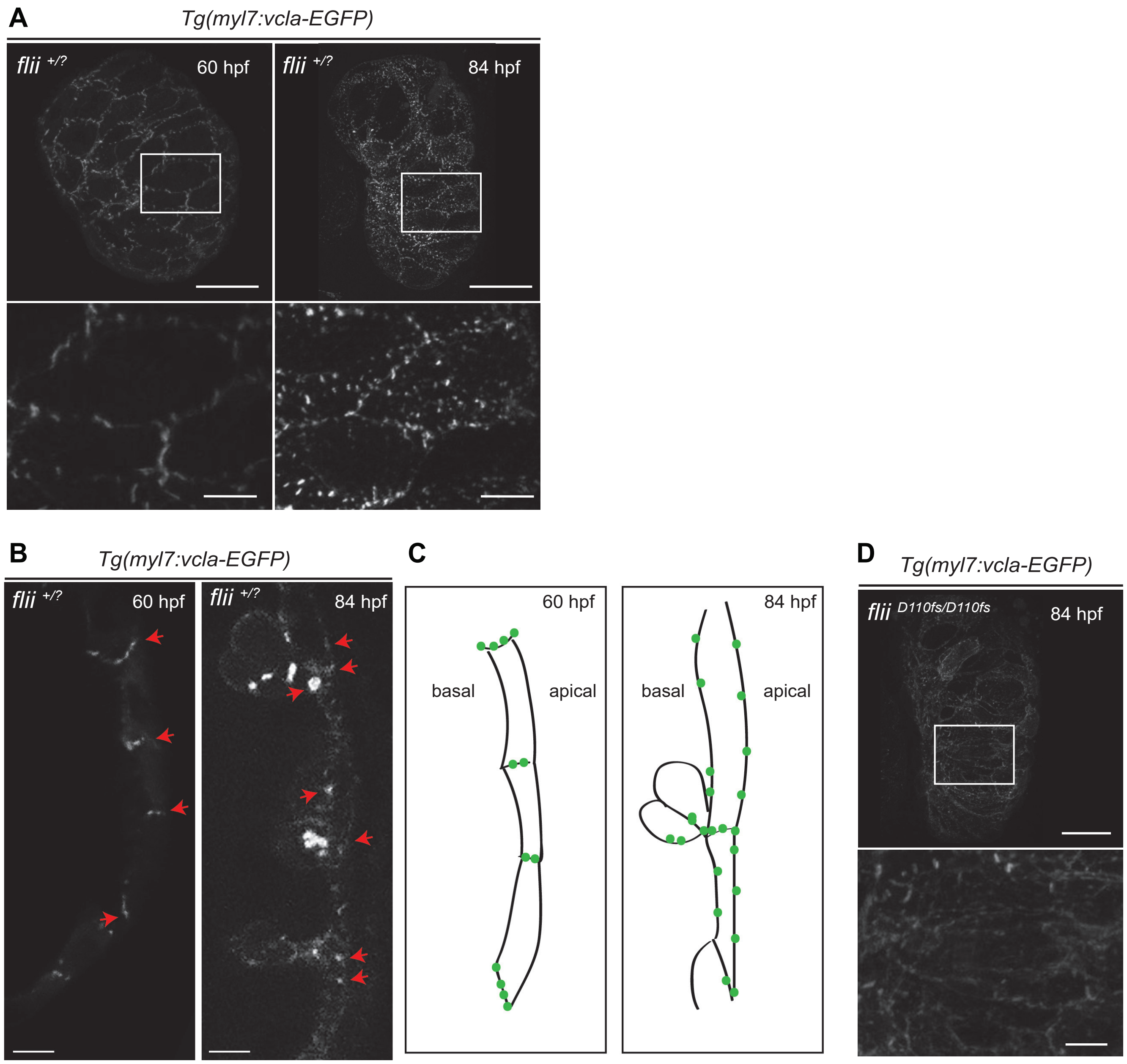

Supplemental Figure 10. Vinculin-EGFP relocalizes during cardiac development. **A**, 3D confocal projections of *Tg(myl7:vcla-EGFP)* sibling hearts at 60 (left panel) and 84 (right panel) hpf. At 60 hpf, vinculin-EGFP foci are restricted to the lateral cardiomyocyte membranes. At 84 hpf, vinculin-EGFP is also present at the apical and basal membranes. This dynamic relocalization is in line with similar findings for other cell adhesion complex components (2). $flii^{+/?}$ siblings 60 hpf n = 5, 84 hpf n = 5; scale bars: projections, 25 µm; magnifications, 5 µm. **B**, Single confocal planes of *Tg(myl7:vcla-EGFP)* sibling ventricles at 60 hpf (left panel) and 84 (right panel) hpf. At 84 hpf, vinculin-EGFP relocalizes and is no longer restricted to the lateral membranes. Red arrows point to vinculin-EGFP foci at lateral cardiomyocyte membranes at 60 hpf and at lateral, apical, and basal membranes at 84 hpf. $flii^{+/?}$ siblings 60 hpf n = 5, 84 hpf n = 5; scale bars: 5 µm. **C**, Cartoon depicting vinculin-EGFP relocalization. **D**, 3D confocal projections of a *Tg(myl7:vcla-EGFP)* $flii^{D110fs/D110fs}$ ventricle at 84 hpf. Note that vinculin-GFP also relocalizes in *flii* mutant animals. n = 5; scale bars: 5 µm;

Supplemental Tabels 1 – 3

Supplemental Table 1. Detailed cardiac findings in patients with bi-allelic *FLII* variants

Patient ID	ECG	Echocardiography At presentation	At last examination
1-II:2	SR, no conduction abnormalities, low QRS voltages, flat T-waves	Severely dilated LV, spherical shape, LVIDd 3.5 cm (z-score 6.3), FS 13%, moderate MR, mild TR	Moderately dilated LV, spherical shape, LVIDd 3.7 cm (z-score 3.3), LVEF 33%, FS 27.6%, mild MR, mild TR
2-II:1	SR	Severely dilated LV, ASD 4 mm mean PG 6 mmHg, LVIDd 3.7 cm (z-score 4), LVEF 23%, FS14 % mild TR	Mildly dilated LV and LA, LVIDd 4.0 cm (z-score 1.3), LVEF 58%, FS 30%, trivial MR, mild PR, trivial TR
3-II:1	SR, no conduction abnormalities, borderline prolonged QTc interval (460 ms), no ST-T changes	Moderately dilated LV, PFO with left to right shunt, LVEF 32%, FS16% LVIDd 3.4 cm (z-score 3), mild MR, mild TR	Moderately dilated LV, dilated LA, intact atrial septum, LVEF 34%, FS14% LVIDd 5.0 cm (z-score2.1), LV apical non-compaction, mild MR , mild TR

Abbreviations: ECG, electrocardiography; FS, fractional shortening; LV, left ventricle; LVEF, left ventricular ejection fraction; LVIDd, left ventricular internal diameter at end diastole; MR, mitral regurgitation; PG, pressure gradient; PR, pulmonary regurgitation; SR, sinus rhythm; TR, tricuspid regurgitation.

Supplemental Table 2. Sequence details of sgRNA target site and ssDNA oligos used in this study

Target	Target-specific guide RNA (5'- 3')	ssDNA oligo (5' – 3')
flii exon 12 (*flii*^{S449fs})	GCTAAAGGGAATGTCAGATG	n.a.
flii exon 27 (*flii*^{R1158W})	TGAAATATGCTAGACTGTTT	ACATGAAATATGCTAGACTCTT**C**TGGTAAGGAAATGAATT
flii exon 30 (*flii*^{R1230C})	GCACCAGGCGCAGTTTTCTG	TGCGCACCAGGCGCAGTTTACAGGGGTTCTCCGTATCCTT

In red: missense variant; in magenta: synonymous nucleotide changes to create novel restriction -enzyme site (underlined); in bold: synonymous nucleotide changes to prevent Cas9 from recutting.

Supplemental Table 3. Genotyping details of *flii* mutant zebrafish

Allele	Method	Primers	Sequence 5’ – 3’	Restriction enzyme
flii ^{S449fs} (exon 12)	Allele specific PCR	Flii-ex11F<br>Flii-ex13R*<br>+<br>Fliiex12Fw_wt_-7bp<br>*or*<br>Fliiex12Fw_mut_- 7bp	TCATTGCAGAACCAGCTACG<br>AATCCAGCTGAGGCTTCTCC<br><br>AGGTGCTRAAGGGAATGTC<br><br>CAGGTGCTRAAGGGAATGTG	n.a.
	Digestion	Flii-ex11F<br>Flii-ex13R	See above	Hpy188I @ 65 ◦C<br>Cuts in wild-type allele
flii ^{R1158W} (exon 27)	Digestion	Flii-ex26F*<br>Flii-in27R	ATTTGAAAGCACAGACAATCAGG<br>ttagcttgacagacctccgc	Hinf1 @ 37◦C<br>Cuts in mutant allele
flii ^{R1230C} (exon 30)	Allele specific PCR	Flii-int29Fc<br>Flii-ex30Rd<br>+<br>Fliiex30Fw_wt<br>*or*<br>Fliiex30Fw_mut	GCCCAAAAGCTCAATCTACG<br>TGTGCTGTTTGTTTTCTGAATGC<br><br>AGGATACGGAGAACCCCAGA<br><br>AGGATACGGAGAACCCCTGT	n.a.
	Digestion	Flii-int29Fc*<br>Flii-ex30Rd	See above	Hpy166II 37◦C<br>Cuts in mutant allele

*primers used for Sanger sequencing

Supplemental References

1. Naganawa Y, Hirata H. Developmental transition of touch response from slow muscle-mediated coilings to fast muscle-mediated burst swimming in zebrafish. *Dev Biol.* Jul 15 2011;355(2):194-204. doi:10.1016/j.ydbio.2011.04.027
2. Cherian AV, et al. N-cadherin relocalization during cardiac trabeculation. *Proc Natl Acad Sci U S A*. Jul 5 2016;113(27):7569-74. doi:1606385113
